# Confinement-induced giant ionic thermovoltage at minimal temperature gradients via series-integrated micro-thermoelectric cells in hierarchical hydrogels

**DOI:** 10.1093/nsr/nwag296

**Published:** 2026-05-23

**Authors:** Mi Fu, Yuwei Yuan, Faqi Hu, Liguo Xu, Dan Zhao, Kan Yue

**Affiliations:** State Key Lab of Luminescent Materials and Devices, Guangdong Provincial Key Laboratory of Functional and Intelligent Hybrid Materials and Devices, Guangdong Basic Research Center of Excellence for Energy and Information Polymer Materials, South China Advanced Institute for Soft Matter Science and Technology, School of Emergent Soft Matter, South China University of Technology, Guangzhou 510640, China; State Key Lab of Luminescent Materials and Devices, Guangdong Provincial Key Laboratory of Functional and Intelligent Hybrid Materials and Devices, Guangdong Basic Research Center of Excellence for Energy and Information Polymer Materials, South China Advanced Institute for Soft Matter Science and Technology, School of Emergent Soft Matter, South China University of Technology, Guangzhou 510640, China; State Key Lab of Luminescent Materials and Devices, Guangdong Provincial Key Laboratory of Functional and Intelligent Hybrid Materials and Devices, Guangdong Basic Research Center of Excellence for Energy and Information Polymer Materials, South China Advanced Institute for Soft Matter Science and Technology, School of Emergent Soft Matter, South China University of Technology, Guangzhou 510640, China; College of Light Chemical Industry and Materials Engineering, Shunde Polytechnic University, Foshan 528333, China; Laboratory of Organic Electronics, Department of Science and Technology, Linköping University, Norrköping 601 74, Sweden; State Key Lab of Luminescent Materials and Devices, Guangdong Provincial Key Laboratory of Functional and Intelligent Hybrid Materials and Devices, Guangdong Basic Research Center of Excellence for Energy and Information Polymer Materials, South China Advanced Institute for Soft Matter Science and Technology, School of Emergent Soft Matter, South China University of Technology, Guangzhou 510640, China; Beijing National Laboratory for Molecular Sciences, Beijing 100190, China

**Keywords:** ionic hydrogels, thermoelectric, short-range diffusion, Seebeck coefficient, hierarchically structured

## Abstract

Harvesting low-grade waste heat, particularly from ubiquitous small temperature fluctuations, requires materials that deliver high voltage outputs under minimal thermal gradients. Conventional ionic thermoelectric (i-TE) hydrogels rely on the global, continuous thermodiffusion of ions (i.e. the Soret effect) across the entire material, which typically yields limited voltages at small temperature differences (Δ*T*). Here, we report a hierarchically structured ionic hydrogel that fundamentally alters this paradigm via a ‘series-integrated micro-thermoelectric cell’ mechanism. By infiltrating a poly(acrylic acid)/sodium acetate (PAA/NaOAc) electrolyte into a directionally freeze-cast polyvinyl alcohol (PVA) scaffold, we construct an anisotropic architecture where dense crystalline PVA domains act as ionic blocking layers perpendicular to the thermal gradient. Under an applied temperature difference, ions undergo short-range thermodiffusion within the confined micro-domains, generating local thermovoltage that accumulates in series. Our hierarchical structured ionic hydrogel yields a record-high ionic Seebeck coefficient of 71.3 mV K^−1^ at minimal temperature gradients (Δ*T* ≤ 2.0 K). Crucially, the thermovoltage exhibits a non-linear saturation behavior at elevated Δ*T*, revealing a different interfacial charge effect in microstructure-confined ion separation. Combined with exceptional mechanical robustness, this work establishes a transformative paradigm shifting from global ion transport to micro-structural series integration, offering a robust material platform for ultra-sensitive thermal sensing and durable self-powered wearable electronics.

## Teaser

A microstructured ionic thermoelectric hydrogel shows a unique nonlinear voltage response through confined ion movement, enabling high thermovoltages from very small temperature differences.

## INTRODUCTION

The efficient harvesting of low-grade waste heat has emerged as a global imperative for the development of sustainable, self-powered electronics [1,2]. While conventional electronic thermoelectric (e-TE) materials are constrained by the coupling of electrical and thermal conductivity, ionic thermoelectric (i-TE) materials have garnered significant attention for their giant thermovoltage [3–5]. Driven by the Soret effect, quasi-solid-state ionic hydrogels exhibit an open-circuit voltage that varies linearly with the temperature gradient. The slope of these fitting lines yields the ionic Seebeck coefficient (*S*_i_), a value that is often orders of magnitude higher than that of traditional electronic thermoelectric materials. To further enhance the performance of i-TE hydrogels, extensive efforts have focused on optimizing the chemical compositions of these materials and tailoring ion-polymer interactions, such as the coordination and hydration effects [6–8], ion-dipole coupling interactions [3,9,10], and hydrogen bonding interactions [11–13]. The prevailing design principle in these studies relies on maximizing the ‘global’ transport of ions, creating unobstructed pathways for ions to diffuse continuously from the hot side to the cold side of the macroscopic material. In the regime of long-range continuous diffusion, the accumulated thermovoltage is typically linear with respect to the temperature difference (Δ*T*) [3,14]. While such strategies have proven effective for large temperature differences, they face an intrinsic limitation when harvesting ubiquitous, minute thermal fluctuations. Consequently, under minimal thermal gradients (e.g. Δ*T* < 2 K), the resulting voltage output is often negligible, severely restricting the sensitivity and applicability of current i-TE materials in scenarios involving subtle environmental heat or human body thermoregulation.

To overcome the limitations of global thermo-driven ion diffusion, one must look beyond chemical composition to the mesoscopic architecture of the materials. In recent years, structural engineering has been employed to enhance ionic transport, typically by aligning channels parallel to the direction of heat flow. For instance, cellulose-based ionic conductors derived from natural wood or aligned porous scaffolds are designed to reduce tortuosity, thereby facilitating faster long-range ion migration [15]. In these conventional anisotropic designs, structural barriers perpendicular to the transport direction are typically regarded as detrimental defects that hinder ion diffusion and degrade conductivity. However, this perspective overlooks the possibility inspired by biological systems. Nature often utilizes compartmentalization and hierarchical barriers to achieve functions that continuous materials cannot. For example, the electric organ of the electric eel (*Electrophorus*) generates high-voltage discharges through the series integration of thousands of microscopic electrocytes stacked in highly ordered arrays, rather than a single cell [16]. Similarly, biological load-bearing tissues like tendons achieve exceptional fatigue resistance through hierarchical, semi-crystalline collagen architectures that dissipate energy across multiple scales [17,18]. These biological precedents suggest that structural ‘barriers’, if designed as functional separators, could serve to compartmentalize a continuous electrolyte into discrete domains.

**Figure 1.**
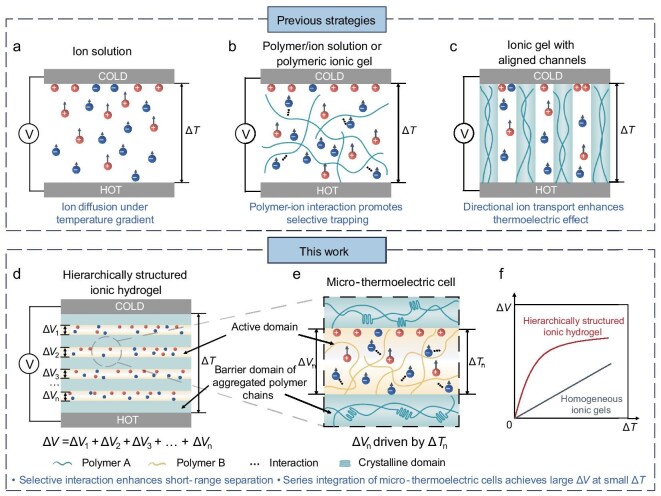
Schematic illustration of the series-integrated micro-thermoelectric cell mechanism. (a–c) Previous strategies relying on long-range ion thermodiffusion: (a) free diffusion of ions in solution driven by the Soret effect; (b) selective ion trapping enabled by polymer-ion interactions in composite ionic gels; (c) directional ion transport facilitated by aligned channels. (d) The hierarchically structured ionic hydrogel consists of alternating active domains and barrier domains, effectively functioning as a series circuit where the total thermovoltage (Δ*V*) is the sum of local voltages (Δ*V*_n_). (e) Magnified view of a micro-thermoelectric cell. Within the active domain, selective interactions between the polymer matrix and ions enhance short-range charge separation, boosting the local potential difference. (f) Proposed Δ*V*–Δ*T* plots showing the distinct behaviors between the thermovoltage output generated by the hierarchically structured ionic hydrogel with the phenomenon of voltage saturation and the conventional linear response in homogeneous ionic gels.

Motivated by this bio-inspired perspective, we propose a fundamental paradigm shift from long-range continuous ion diffusion to a ‘series-integrated micro-thermoelectric cell’ mechanism (Fig. 1). Using a cation-dominated p-type system as a representative example, in simple ion solutions (Fig. 1a), cations and anions diffuse freely, often resulting in low thermovoltage due to similar mobilities. Improvements have been made by introducing polymers (Fig. 1b) to selectively trap specific ions, or by constructing aligned channels (Fig. 1c) to enhance ion transport. However, these traditional ionic thermoelectric systems primarily rely on the continuous long-range diffusion of ions driven by a large Δ*T*. In contrast, this work proposes to employ a hierarchically structured ionic hydrogel configured for energy harvesting in the perpendicular direction of the aligned electrolyte domains (Fig. 1d). Fundamentally distinct from the macroscopic stacking of independent cells which merely divide the total applied Δ*T* without enhancing the intrinsic material performance [19], our approach relies on a microscopic topological arrangement. In this model, the ionic gel is effectively discretized into an array of microscopic active domains separated by semi-blocking barrier domains (Fig. 1e). When a temperature gradient is applied, ions within each confined active domain undergo short-range thermodiffusion confined by the barrier domains, inducing a local charge separation and a corresponding unit voltage Δ*V*_n_.

Crucially, a distinct signature of this confinement mechanism, which differentiates it from the standard Soret effect, is the phenomenon of voltage saturation (Fig. 1f). We postulate that ions cannot cross the boundaries of each microdomain due to the blocking of crystalline walls, which imposes a no-flux boundary condition and a finite-space mass conservation constraint. In other words, each micro-cell contains only a limited number of mobile ions, and the thermally driven ion redistribution can only occur within this confined volume. As Δ*T* increases, ions accumulate near one side of the micro-cell and deplete near the other side, generating an internal electric field. At equilibrium, the thermodiffusion driving force is balanced by the concentration gradient and the induced electric field, so that the system reaches a thermodynamic steady state. Because the total number of ions available for redistribution inside each micro-cell is finite, the amount of charge separation is also finite, which means that each micro-cell has a maximum charge-storage capacity and therefore a maximum local thermovoltage (Δ*V*_total_ = ΣΔ*V*_n_).

Herein, we experimentally realize this design by fabricating a hierarchically structured ionic hydrogel that integrates a poly(acrylic acid)/sodium acetate (PAA/NaOAc) electrolyte into a directionally freeze-cast polyvinyl alcohol (PVA) skeleton. This architecture is specifically engineered to function as a series of micro-thermoelectric cells. The directional freeze-casting creates a honeycomb-like array of microchannels, while a subsequent salting-out treatment induced by high NaOAc content densifies the PVA walls, transforming them from porous scaffolds into highly crystalline, semi-insulating barriers perpendicular to the thermal gradient. This structural densification is critical for preventing ion leakage between cells while maintaining the series connectivity of the electric field. As a result, our hierarchically structured hydrogel delivers a record-high ionic Seebeck coefficient of 71.3 mV K^−1^, specifically triggered by minimal temperature differences (Δ*T* ≤ 2 K). Consistent with our theoretical model, we observe a distinct voltage saturation plateau at elevated temperature gradients, confirming the dominance of the confinement-induced integration mechanism over global diffusion. In this regime, the voltage response no longer follows a linear trend, and the generated potential is thus more broadly referred to as a thermopower. Furthermore, the dense, anisotropic aggregation of the polymer skeleton endows the hydrogel with exceptional mechanical properties, including a fracture toughness of 95.9 MJ m^−3^, mimicking the robustness of natural load-bearing tissues. This work not only offers insights into the structural design principle of high-performance i-TE hydrogels, but also provides a material platform for ultra-sensitive thermal sensing and durable wearable thermoelectric devices.

## RESULTS AND DISCUSSION

### Design and fabrication of the anisotropic ionic hydrogels

**Figure 2.**
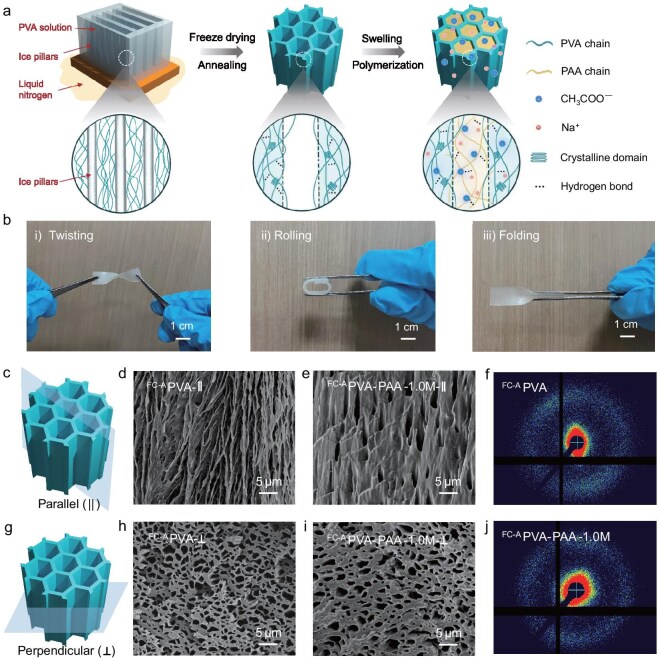
(a) Schematic illustration of the fabrication process of the ^FC-A^PVA-PAA-NaOAc ionic hydrogels. (b) Photographs of the ^FC-A^PVA-PAA-1.0 M ionic hydrogels (10 × 30 × 1.2 mm^3^) captured in the state of i) twisting, ii) rolling and iii) folding. (c) Schematic illustration of the ice-template direction (||), with scanning electron microscopy (SEM) images of (d) ^FC-A^PVA and (e) ^FC-A^PVA-PAA-1.0 M hydrogels along this direction. (f) SAXS 2D patterns for ^FC-A^PVA hydrogels. (g) Schematic illustration of the direction perpendicular (⊥) to the ice-template, with SEM images of (h) ^FC-A^PVA and (i) ^FC-A^PVA-PAA-1.0 M hydrogels. (j) SAXS 2D patterns for ^FC-A^PVA-PAA-1.0 M ionic hydrogels.

As illustrated in Fig. 2a, our fabrication strategy of the hierarchically structured anisotropic ionic hydrogel integrates directional freeze-casting with *in-situ* photo-polymerization to construct a hierarchical PVA-PAA-NaOAc composite tailored for the micro-cell integration mechanism. PVA was selected as the polymer matrix in this study because of its abundant hydrogen-bonding sites and the unique physical crosslinking via partial crystallization [20]. First, a PVA solution was transferred into a customized mold and put in contact with liquid nitrogen to induce the directional freezing. During this process, ice crystals grow vertically along the temperature gradient, expelling PVA chains into the interstitial spaces and inducing their alignment into ordered, lamellar microstructures [21]. Subsequent freeze-drying removes the ice template, yielding a highly porous PVA aerogel skeleton with vertically aligned microchannels. This anisotropic skeleton not only serves as the primary load-bearing framework [18], but also establishes the structural foundation for the barrier domains. Next, the PVA scaffold was infiltrated with a precursor solution containing acrylic acid (AA) monomer, photoinitiator, and electrolyte NaOAc. Upon UV irradiation, the AA monomer undergoes *in situ* photo-polymerization within the confined microchannels, forming a secondary PAA network that interpenetrates the PVA skeleton.

Crucially, the introduction of NaOAc serves a dual purpose. On one hand, it induces the Hofmeister ‘salting-out’ effect [22], which promotes the aggregation of PVA chains and the formation of dense crystalline domains (or crystallites). This transforms the loose network into a compact architecture, effectively solidifying the barrier required to compartmentalize the electrolyte. On the other hand, the acetate (CH_3_COO^−^) anions interact with the PVA/PAA network via hydrogen-bonding interactions, which amplifies the mobility disparity between Na^+^ and CH_3_COO^−^ and yields an enhanced thermoelectric effect [13]. Consequently, we hypothesize that this specific architecture, which is comprised of dense crystalline PVA walls acting as the barrier domains and PAA/NaOAc-filled channels serving as the active domains, can physically realize our proposed structure of the series-integrated micro-thermoelectric cells (Fig. 1d).

By adjusting the content of NaOAc, different ionic hydrogels were fabricated and designated as ^FC-A^PVA-PAA-*x*M (*x* = 0.3, 0.5, 0.7, 1.0, 1.3), where ‘FC’ denotes the directional freezing process, ‘A’ denotes the annealing process, and ‘*x*M’ indicates the concentration of NaOAc aqueous solutions. Thermogravimetric analysis (TGA) confirms the successful incorporation of NaOAc into the composite hydrogels, as evidenced by the variation in residual mass (Fig. S1). The phase behavior and network structure were further probed using differential scanning calorimetry (DSC). As shown in Fig. S2, the ionic hydrogels exhibit two distinct glass transition temperatures (*T*_g_). A lower transition in the range of 45–65°C corresponds to the PAA network, and a higher transition around 75°C is attributed to the PVA skeleton [21]. The presence of these two separate *T*_g_ values confirms the successful formation of a phase-separated microstructure rather than a single homogeneous phase. Furthermore, Fourier-transform infrared (FT-IR) spectroscopy provides evidence for specific molecular interactions within the composite. The characteristic peak at 1702 cm^−1^, assigned to the C=O stretching vibration of the carboxyl group (−COOH) in PAA, shifts to 1712 cm^−1^ upon formation of the double network and further to 1714 cm^−1^ following NaOAc incorporation. Concurrently, the peak corresponding to the symmetrical C–O stretching in PVA shifts from 1090 cm^−1^ to 1099 cm^−1^. These significant spectral shifts substantiate the existence of strong hydrogen-bonding interactions, both between the distinct polymer chains and between the polymer networks and CH_3_COO^−^ ions (Fig. S3) [23–25].

Macroscopically, the resulting PVA-PAA composite hydrogels exhibit excellent flexibility and resilience, capable of being readily twisted, rolled, or folded into arbitrary shapes without fracture (Fig. 2b). Microscopically, scanning electron microscopy (SEM) reveals the highly anisotropic architecture of both the ^FC-A^PVA scaffold and the ^FC-A^PVA-PAA-1.0 M ionic hydrogel. When viewed parallel to the freezing direction (Fig. 2c–e), both materials display aligned channel structures. However, in the cross-sectional view perpendicular to the ice growth (Fig. 2g–i), the freeze-casting process induces a highly ordered, honeycomb-like cellular array in the PVA skeleton. Upon the infiltration of PAA and NaOAc, the ^FC-A^PVA-PAA-1.0 M hydrogel retains this highly oriented framework parallel to the ice-template direction (||). Notably, the composite hydrogel exhibits a denser morphology with fewer observable pores compared to the pristine PVA scaffold, indicating the effective filling of the microchannels by the secondary PAA network. Furthermore, the pore size distribution analysis reveals that the ^FC-A^PVA-PAA-1.0 M ionic hydrogel possesses an average micro-cell diameter (d) of approximately 1.29 μm (Fig. S4). Based on this microscopic dimension and the macroscopic thickness of our sample (*L* ≈ 1.2 mm), we can estimate that there are approximately *n* = *L/d* ≈ 930 micro-cells connected in series along the direction of the temperature gradient. Given the macroscopic maximum thermal voltage of Δ*V* ≈ 140 mV under a macroscopic temperature difference of Δ*T* = 2 K, we can roughly estimate that the local voltage drop across each individual micro-cell is approximately Δ*V_n_* ≈ 0.15 mV, and the local temperature difference across each cell is about Δ*T_n_* ≈ 0.002 K. This semi-quantitative estimation effectively demonstrates how the macroscopic thermoelectric output is cumulatively generated by the microscopic porous units, further highlighting the rationale and innovation of our anisotropic structural design. The anisotropic alignment of the crystallites within the polymer matrix is further corroborated by small-angle X-ray scattering (SAXS) patterns (Fig. 2f and j), which display distinct orientation features consistent with the freeze-casting and annealing processes [21]. Additionally, confocal microscopy images provide complementary visualization, confirming the ordered microstructure of the ionic hydrogel throughout the bulk material (Fig. S5). As shown in Fig. 2e and i, although the actual microstructure of the ^FC-A^PVA-PAA hydrogel presents a complex 3D honeycomb-like morphology [21] rather than the idealized 1D layered stack as depicted in Fig. 1, the fundamental mechanism of the series-integrated microcell model remains valid. When a temperature gradient is applied to the perpendicular direction of the lamellae or the channels, ionic charge carriers traversing the porous network encounter a sequential series of potential barriers (polymer walls) and conductive regions (electrolyte domains), which is topologically equivalent to the proposed series circuit model [21].

### Microstructure of the ^FC-A^PVA-PAA-NaOAc ionic hydrogels

**Figure 3.**
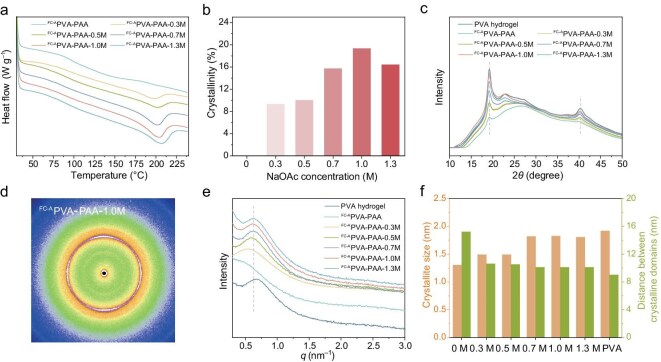
(a) DSC curves of the ^FC-A^PVA-PAA-NaOAc ionic hydrogels with different NaOAc contents. (b) Corresponding crystallinity values of the ^FC-A^PVA-PAA-NaOAc ionic hydrogels with different NaOAc contents. (c) WAXS profiles of the PVA hydrogels and the ^FC-A^PVA-PAA-NaOAc ionic hydrogels with different NaOAc contents. (d) WAXS patterns for the ^FC-A^PVA-PAA-1.0 M ionic hydrogel. (e) SAXS profiles of the PVA hydrogels and the ^FC-A^PVA-PAA-NaOAc ionic hydrogels with different NaOAc contents. (f) Estimated average size of the crystallites and average distance between adjacent crystallites of the ^FC-A^PVA-PAA-NaOAc ionic hydrogels with different NaOAc contents.

To elucidate the structural origins of the barrier domains, we systematically investigated the microstructural evolution of the hydrogels with different concentrations of the salting-out agent (NaOAc). Differential scanning calorimetry (DSC) thermograms (Fig. 3a) illustrate the thermal behavior of ^FC-A^PVA-PAA- NaOAc ionic hydrogels over the range of 50–220°C. Except for the salt-free sample, all ionic hydrogels display a prominent endothermic peak centered around 200°C, attributed to the melting of PVA crystallites. Quantitative integration of these melting endotherms reveals that the ^FC-A^PVA-PAA-NaOAc hydrogels exhibit a high degree of crystallinity (Fig. 3b and Table S1). The crystallinity follows a bell-shaped trend, peaking at 19.3% for the 1.0 M NaOAc formulation.

This conclusion is further substantiated by wide-angle X-ray scattering (WAXS) analysis (Fig. 3c). All hydrogels show characteristic diffraction peaks at 2θ values of 19.2°, 23.0°, and 40.3°, corresponding to the (${\mathrm{10\bar{1}}}$), (200), and (102) crystallographic planes of semicrystalline PVA, respectively [26,27]. Consistent with thermal analysis, the ^FC-A^PVA-PAA-1.0 M ionic hydrogel displays the sharpest and most intense diffraction maxima, indicating the highest degree of crystallinity. This is visually corroborated by the distinct, high-intensity diffraction arcs observed in the 2D WAXS patterns (Fig. 3d), which reflect the anisotropic orientation of the crystallites. We further quantified the semicrystalline architecture by calculating the average crystallite size (*D*) using the Scherrer equation and the long period (average distance between adjacent crystallites, *L*) from SAXS profiles via Bragg’s law [21,28]. The SAXS scattering profiles (Fig. 3e) exhibit analogous salt content-dependent behaviors and the evolution of crystallite size *D* and inter-domain spacing *L* as a function of salt content is compiled in Fig. 3f and Table S2. These results elucidate a competitive mechanism governing microstructure formation. Initially, the infiltration of the amorphous PAA network disrupts the native order of the PVA scaffold. However, the introduction of salt ions triggers the Hofmeister effect [22], which dehydrates the PVA chains and drives their aggregation, thereby progressively restoring and enhancing crystallinity [29]. Nevertheless, an optimal salt content exists; at NaOAc concentrations exceeding 1.0 M, the excessive salting-out effect induces overly rapid and kinetic-controlled aggregation of PVA chains. This rapid precipitation precludes the reorganization of chains into regular lattice structures, leading to a subsequent decline in overall crystallinity. This coarsening of crystallites is critical for enhancing the barrier domains within the polymer network and reinforcing its mechanical properties [30,31].

### Ionic thermoelectric performance of the ^FC-A^PVA-PAA-NaOAc ionic hydrogels

**Figure 4.**
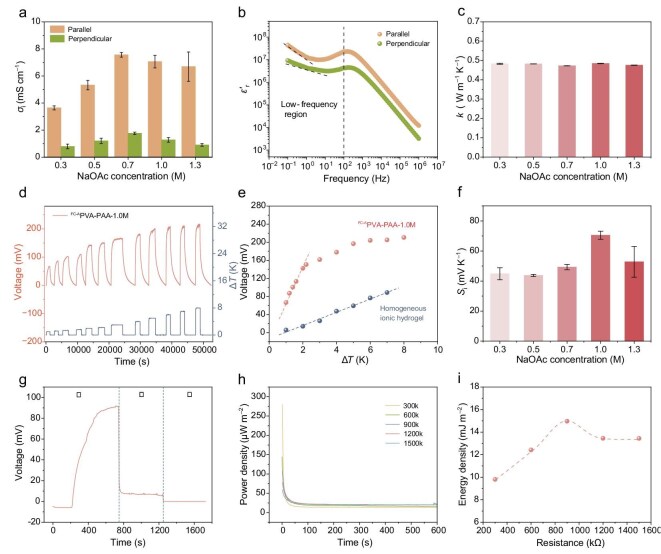
(a) Ionic conductivity in different directions of ^FC-A^PVA-PAA-NaOAc ionic hydrogels with different NaOAc contents. (b) Frequency dependence of the real part ofε’_r_ for the ^FC-A^PVA-PAA-1.0 M ionic hydrogels measured in parallel and perpendicular directions. (c) Thermal conductivity of ^FC-A^PVA-PAA-NaOAc ionic hydrogels with different NaOAc contents. (d) Real-time response of the generated thermovoltage and the applied temperature difference for the ^FC-A^PVA-PAA-1.0 M ionic hydrogel. (e) Variation of thermovoltage with the temperature difference of the ^FC-A^PVA-PAA-1.0 M ionic hydrogel and the homogeneous PEO-PAA-NaOAc ionic hydrogel. (f) Seebeck coefficient values of the ^FC-A^PVA-PAA-NaOAc ionic hydrogels with different NaOAc contents measured at Δ*T* ≤ 2 K. (g) Operating mode of the ionic thermoelectric generators (ITEGs) with the ^FC-A^PVA-PAA-1.0 M ionic hydrogels. (h) Power density profiles of external loads with different resistances connected to an ITEG. (i) Plot of total charging and discharging energy density versus the resistance of the external load measured for 10 min.

The incorporation of NaOAc serves as the source of mobile charge carriers, which dissociate into sodium ions and acetate anions and migrate through the polymer matrix via coordination-dissociation hopping mechanisms [32]. We quantified the ionic conductivity (*σ_i_*) using electrochemical impedance spectroscopy (EIS), deriving the bulk resistance (*R*) from the high-frequency intercept of the Nyquist plots (Figs S6 and S7). The impact of salt content on *σ_i_* is governed by the interplay between carrier density and carrier mobility. As illustrated in Fig. 4a, *σ_i_* initially increases with NaOAc content due to the increased population of charge carriers. However, beyond a critical concentration, the pronounced salting-out effect induces excessive polymer chain aggregation and crystallization [29]. This densification reduces the free volume available for ion transport and increases the tortuosity of the conduction pathways, leading to a subsequent decline in conductivity (Tables S3 and S4). Crucially, the ionic conductivity exhibits significant anisotropy (Fig. 4a). In the parallel orientation (||), the aligned continuous microchannels act as ‘ion highways’, minimizing geometric obstruction and reducing tortuosity [7,15]. Consequently, the ^FC-A^PVA-PAA-1.0 M hydrogel achieves a peak *σ_i_* of 7.56 mS cm^−1^ in the parallel direction, which is over fourfold higher than that measured in the perpendicular direction (⊥). This result confirms that PVA functions as a dense barrier that significantly hinders ionic transport.

To eliminate the geometric influence of different sample dimensions and probe the intrinsic dielectric anisotropy, the frequency-dependent relative permittivity (*ε’_r_*) spectra were derived from the impedance data (Fig. 4b). Notably, the *ε’_r_* in the parallel direction is consistently higher than that in the perpendicular direction across the measured frequency range. In the perpendicular direction, ions encounter dense insulating barriers of semicrystalline PVA lamellae, which effectively act as series-connected capacitors, thereby reducing the overall effective permittivity [33]. Conversely, the parallel direction offers more continuous ionic channels, facilitating stronger macroscopic polarization. This dielectric anisotropy provides compelling evidence for the structural orientation, supporting the mechanism where ions are spatially confined by barriers in the perpendicular direction.

Furthermore, the frequency dependence in the low-frequency region (<100 Hz) reveals distinct polarization dynamics. The *ε′_r_* parallel to the channels (yellow curve) exhibits a sharp increase as frequency decreases. This is a typical signature of space charge polarization induced by the long-range migration and accumulation of ions [34]. In stark contrast, the ε′_r_ perpendicular to the channels (green curve) displays a much flatter variation in this low-frequency regime. This plateau-like behavior suggests that the charge storage mechanism in the perpendicular direction is governed by localized, short-range ionic responses rather than long-range diffusion [35]. The ions are confined within micro-domains and respond rapidly; once the frequency is low enough to fully activate this intra-domain polarization, the capacitance saturates and remains relatively stable. This unique intrinsic polarization characteristic confirms the absence of long-range ionic continuity in the perpendicular direction, which is the key prerequisite for establishing the ‘series-connected micro-thermoelectric cells’ and achieving the giant thermoelectric output. Additionally, the thermal conductivity (*κ*) was measured via the transient hot-wire method. Regardless of salt content, all samples maintain a low *κ* of approximately 0.45 W m^−1^ K^−1^ (Fig. 4c and Table S5), a desirable feature that helps sustain stable temperature gradients across the ionic hydrogel materials [36,37].

The ionic Seebeck coefficients *S_i_* of the ^FC-A^PVA-PAA-NaOAc hydrogels were measured using a custom-designed sandwich-type setup (Fig. S8). Figure 4d and e presents the variation of thermovoltage as a function of Δ*T* for the ^FC-A^PVA-PAA-1.0 M ionic hydrogels in the perpendicular direction. Specifically, the *S_i_* is defined here as the slope of the linear fit of the open-circuit voltage (Δ*V*) against the temperature difference Δ*T* [4]. It is important to note that due to the saturation of the confinement-induced thermovoltage, the reported constant *S_i_* is valid primarily within the low temperature difference range (linear regime, Δ*T* ≤ 2 K) (Fig. 4e and Fig. S9). For larger temperature differences (Δ*T* > 2 K), the voltage response deviates from linearity, and the generated potential is more broadly referred to as the thermopower. This saturation behavior strongly validates our proposed series-integrated micro-thermoelectric cell mechanism with confined ion diffusion. Unlike the continuous long-range diffusion where charge separation occurs at macroscale distance, the ions in our system are confined within discrete micro-domains separated by the crystalline PVA barriers. The resulting internal electric fields rapidly counteract further ionic transport, causing each cell to reach electrochemical equilibrium at small Δ*T*.

It is worth noting that the deviation from linearity at Δ*T* > 2 K is not an artifact of the measurement setup (e.g. thermal convection in the thin sandwich structure). To verify this, we performed a control experiment using a PEO-PAA-NaOAc hydrogel with a similar thickness (∼1.2 mm) under the same experimental conditions. In great contrast, the control group of a homogeneous PEO-PAA-NaOAc ionic hydrogel exhibits a linear thermovoltage response across the entire measured Δ*T* range (Fig. 4e and Fig. S10), consistent with the classical Soret effect where ions undergo continuous long-range thermodiffusion without geometric confinement [14]. This comparison confirms that the giant *S_i_* is driven by the series integration of local, short-range charge separations rather than global ion migration. Moreover, the geometric confinement of the PVA skeleton dictates the voltage saturation behavior, while the chemical environment determines the magnitude of the thermovoltage. As evidenced by the control experiment on the PVA-1.0 M system (Fig. S11), the voltage saturation persists even without PAA, suggesting the blocking effect of the semi-crystalline PVA walls. However, the presence of PAA network is crucial for achieving a high Seebeck coefficient, as the hydrogen bonding interactions between PAA and NaOAc significantly amplify the intrinsic Soret effect before saturation occurs [11,13]. To further verify the robustness of the confinement effect, the temporal stability of the thermovoltage was evaluated. As shown in Fig. S12, the Δ*V* maintains a stable plateau for over 2 hours under a constant temperature difference of 6 K. The absence of obvious voltage decay indicates that the semi-crystalline PVA walls serve as effective barriers with negligible ion leakage, successfully sustaining the confinement-induced potential gradient over long periods of several hours.

Furthermore, the ionic Seebeck coefficients were extracted by linearly fitting the data within this restricted Δ*T* range (2 K). As shown in Fig. 4f, the *S_i_* of the ^FC-A^PVA-PAA-NaOAc ionic hydrogels measured at small Δ*T* (2 K) exhibits a non-monotonic dependence on salt content, reaching a maximum at 1.0 M NaOAc. The initial rise is attributed to the enhanced number of mobile cations participating in the Soret effect. However, with higher ionic strengths, the Debye screening length of the electrical double layer decreases significantly. This screening effect weakens the electrostatic coupling between the polymer network and the mobile ions, thereby diminishing the ion-selection capability of the matrix and reducing the net thermovoltage [38]. Remarkably, the optimized ^FC-A^PVA-PAA-1.0 M hydrogel delivers a record-high ionic Seebeck coefficient at a small Δ*T* ≤ 2 K. Based on five independent parallel measurements (Fig. 4d and Fig. S13), we obtained a reproducible average *S_i_* of 71.3 mV K^−1^ (Fig. 4f). This value is significantly higher than recent representative values reported for p-type ionic thermoelectric materials [39–42].

To evaluate whether interfacial redox reactions may contribute to the measured thermal voltage, cyclic voltammetry was carried out using a symmetric two-electrode Pt/hydrogel/Pt configuration under isothermal conditions. As shown in Fig. S14, the CV curves exhibit a predominantly quasi-rectangular shape within the practical device operating voltage window (< 0.25 V), indicating that electric-double-layer charging is the dominant process in this range. Although an increase in current is observed near the edge of the scan window, suggesting the possible onset of electrochemical side reactions at higher applied voltages, this potential range is outside the actual thermoelectric operating window and is therefore unlikely to contribute significantly to the measured thermovoltage [43–46].

Unlike electronic thermoelectric generators that operate continuously, ionic thermoelectric generators (ITEGs) function as thermally chargeable capacitors based on the Soret effect [15,44]. To demonstrate the practical energy harvesting capability, we fabricated an ITEG device using the optimized ^FC-A^PVA-PAA-1.0 M ionic hydrogel. As shown in Fig. 4g, the operation cycle consists of three distinct stages. In Stage I of thermal charging, under an open-circuit condition with a temperature difference of Δ*T* = 1.5 K, a thermovoltage of approximately 90 mV is established due to the thermodiffusion and accumulation of Na^+^ cations at the cold side and CH_3_COO^−^ anions at the hot side, respectively. In Stage II of electrical discharge, connecting the ITEG to an external load allows the stored charge to dissipate, resulting in a rapid voltage decay as the electrical work is performed. In Stage III of self-recovery, the device is short-circuited under isothermal conditions to promote ion back-diffusion and charge recombination, which resets the ion distribution and restores the device to its initial state for the next cycle [47]. To evaluate the discharge characteristics, various external loads ranging from 300 to 1500 kΩ were applied to the ITEG. Figure 4h illustrates the corresponding output power densities measured during Stage II. The total accumulated energy was determined via the integral formula *∫E = ∫IVdt*, with *I, V*, and *t* representing the load current, the voltage, and the output time, respectively [19]. Based on this calculation, Fig. 4i summarizes the energy densities achieved over a 10-minute discharge at a Δ*T* of 1.5 K across the different loads. Taking the data using a 900 kΩ load as an example, the ITEG demonstrates stable energy output for a duration of 2 h (Fig. S15).

### Mechanical properties of the ^FC-A^PVA-PAA-NaOAc ionic hydrogels

**Figure 5.**
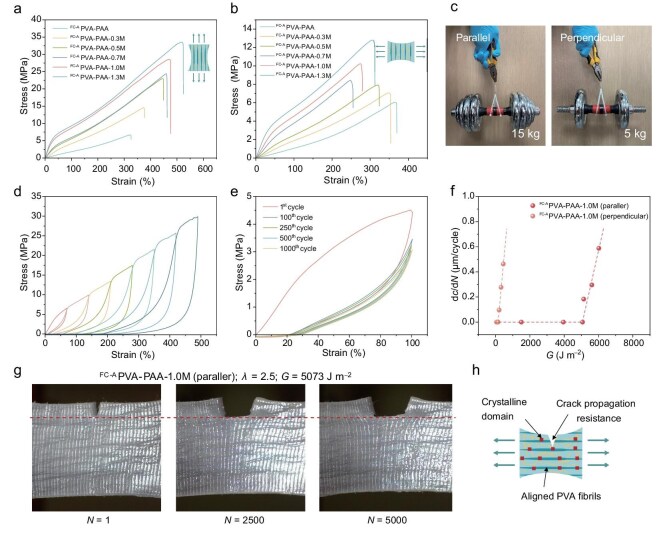
(a and b) Representative stress–strain curves of the ^FC-A^PVA-PAA-NaOAc ionic hydrogels with different NaOAc contents in (a) parallel and (b) perpendicular directions. (c) Images demonstrating the remarkably high strength of the as-prepared hydrogels along the alignment direction, by sustaining a dumbbell (15 kg) with over 20 000 × its own weight. It can also support a 5 kg dumbbell in the unaligned direction. (d) Continuous tensile loading–unloading stress–strain curves of the ^FC-A^PVA-PAA-1.0 M ionic hydrogels with incremental strain. (e) Cyclic stress–strain curves of the ^FC-A^PVA-PAA-1.0 M ionic hydrogels over 1000 cyclic loads. (f) Crack extension per cycle d*c*/d*N* versus applied energy release rate *G* for the ^FC-A^PVA-PAA-1.0 M ionic hydrogel in both parallel (||) and perpendicular (⊥) directions. (g) Validation of the parallel fatigue threshold of 5.073 kJ m^−2^ for the ^FC-A^PVA-PAA-1.0 M ionic hydrogel using the single-notch test at cycle numbers of 1, 2500, and 5000. The streaks observed on the ionic hydrogels are imprinted paths from the 3D-printed mold used during the freeze-casting processes. (h) Schematic diagram of the ionic hydrogel used for testing the horizontal fatigue threshold and the mechanism of crack propagation passivation.

Attributable to the aligned PVA skeleton, the ^FC-A^PVA-PAA-NaOAc ionic hydrogels exhibit pronounced mechanical anisotropy. We define the testing configuration where the tensile load is applied along the direction of fiber alignment as the ‘parallel’ orientation (||), and the configuration orthogonal to the alignment as the ‘perpendicular’ orientation (⊥) (Fig. S16). Figure 5a and b presents the representative stress-strain curves for hydrogels prepared with varying NaOAc contents. As clearly observed in both orientations, the tensile strength, toughness, and Young’s modulus increase progressively with increasing salt content. We quantified the degree of mechanical anisotropy using the Anisotropy Ratio (*H*/*V* ratio), defined as the ratio of a specific mechanical property measured in the parallel direction to that in the perpendicular direction. Specifically, the optimized composite ionic hydrogel achieves a remarkable tensile strength of 33.6 MPa, a toughness of 95.9 MJ m⁻^3^, and a Young’s modulus of 22.2 MPa (Fig. S17). The excellent mechanical property is further corroborated by rheological measurements (Fig. S18). This substantial mechanical reinforcement stems from the ‘salting-out’ effect induced by the high content of acetate ions, which promotes the densification of PVA chains and the formation of crystallites [29].

Interestingly, while the fracture strength in the perpendicular direction increases monotonically with salt concentration, the elongation at break displays a non-monotonic trend: it initially decreases before subsequently recovering. We hypothesize that at lower salt concentrations, the sparse distribution of crystallites leads to polymer chain contraction, which restricts network extensibility and induces brittle fracture. Conversely, at elevated salt concentrations, the formation of a dense and ordered hydrogen-bonding network enables a toughening mechanism involving the ‘stick-slip’ extraction of PVA chains from crystallites. This process effectively dissipates energy, necessitating higher strain energy for deformation and thereby restoring ductility [20]. The exceptional load-bearing capacity of the material is visually demonstrated in Fig. 5c. A thin rectangular strip (40 × 5 × 1.2 mm^3^) can lift a 15-kg weight in the parallel direction, corresponding to a stress of > 25 MPa. Even in the perpendicular direction, the ionic hydrogel readily sustains a 5-kg load without failure.

Long-term fatigue resistance under cyclic loading represents the critical bottleneck for the practical deployment of i-TE hydrogels in wearable electronics [48]. Figure 5d displays the stress-strain curves of the ^FC-A^PVA-PAA-1.0 M hydrogel under escalating strain amplitudes in a cyclic tensile test. Each loading-unloading cycle exhibits a pronounced hysteresis loop, characteristic of viscoelastic energy dissipation. A significant Mullins effect is observed, indicating that the initial loading progressively disrupts the sacrificial non-covalent interactions and disentangles polymer chains, leading to microstructural reconfiguration. Concurrently, the dissipated energy stabilizes after the initial cycles (Fig. S19), suggesting that the rupture and reformation of hydrogen bonds attain a dynamic equilibrium [49].

Under a fixed 100% strain for 1000 cycles (Fig. 5e), the ^FC-A^PVA-PAA-1.0 M ionic hydrogel displays substantially large hysteresis areas. This superior energy dissipation capacity stems from the robust intermolecular interactions within the highly oriented network. During deformation, the aligned crystallites act as physical crosslinks that facilitate extensive chain slippage and reorientation, thereby dissipating more energy while maintaining structural integrity [49]. The ^FC-A^PVA-PAA-1.0 M ionic hydrogel also manifests a distinct Mullins effect, indicating irreversible structural transformation during cyclic loading. This behavior arises from the strain-adaptive nature of salting-out-induced PVA crystallites, which resist network deformation at low strains while progressively unfolding at higher strains to enable continuous energy dissipation [50,51]. After approximately 100 cycles, the stress-strain curves nearly converge and the dissipated energy stabilizes, signifying that hydrogen bond breakage and reformation within the polymer network have reached dynamic equilibrium (Fig. 5e and Fig. S20).

To rigorously quantify the fatigue resistance, we measured the fatigue threshold (*Γ*_0_), the critical energy release rate below which cracks do not propagate, using the single-notch test method (Fig. S21). As shown in Fig. 5f, the ^FC-A^PVA-PAA-1.0 M ionic hydrogel demonstrates anisotropic fatigue resistance with a substantial *Γ*_0__||_ of 5073 J m^−2^ and *Γ*_0__⊥_ of 150 J m^−2^. This dramatic enhancement is attributed to the synergistic effects of highly aligned nanofibrillar architecture and elevated crystallinity. These fatigue thresholds were validated by 5000-cycle tests at energy release rates of 1024 J m^−2^ and 5073 J m^−2^, with no detectable crack extension observed (Fig. 5g). As schematically shown in Fig. 5h, during deformation, when a crack attempts to propagate perpendicular to the fiber alignment, the stress is effectively delocalized by the dense array of oriented crystallites, which further effectively arrests the crack propagation via a ‘crack pinning’ mechanism [52].

## CONCLUSION

In summary, we have successfully validated a series-integrated micro-thermoelectric cell mechanism that overcomes the intrinsic limitations of global ion diffusion at minimal temperature gradients. By infiltrating a PAA/NaOAc electrolyte into a directionally freeze-cast PVA scaffold, we engineered an anisotropic hierarchical hydrogel where dense semicrystalline PVA barriers compartmentalize ions into discrete active micro-domains. This architecture enables the series accumulation of local thermovoltage generated by short-range diffusion, yielding a record-high ionic Seebeck coefficient of 71.3 mV K^−1^ specifically at small temperature differences (Δ*T* ≤ 2 K). Simultaneously, the aligned and densified PVA skeleton, reinforced by the salting-out effect, resolves the longstanding trade-off between mechanical durability and electrochemical performance. The resulting material exhibits an unprecedented combination of properties, including a tensile strength of 33.6 MPa, a fracture toughness of 95.9 MJ m^−3^, and a fatigue threshold of 5073 J m^−2^. This work represents a conceptual leap from optimizing global ion transport to engineering micro-structural series integration. Furthermore, we emphasize that the primary advantage of this material is for voltage-sensitive applications (like triggering gates in sensors) under minimal temperature differences, where high open-circuit voltage is more critical than maximum power output [53]. This strategy provides a decisive solution for harvesting ubiquitous low-grade heat, specifically in the subtle range of physiological temperature gradients. Given its combination of extreme sensitivity and exceptional fatigue resistance, we expect that our hydrogel might create exciting opportunities for ultrasensitive thermal sensing applications in the field of wearable devices for healthcare monitoring [54].

## MATERIALS AND METHODS

### Fabrication of hierarchical ionic hydrogels

The anisotropic ^FC-A^PVA-PAA-NaOAc hydrogels were fabricated via a sequential strategy involving directional freeze-casting, thermal annealing, and *in-situ* photo-polymerization. Briefly, a degassed aqueous PVA solution (10 wt.%) was directionally frozen on a liquid nitrogen-cooled copper stage to induce vertically aligned ice templating. Following lyophilization (−80°C) and annealing at 100°C to crystallize the skeleton, the resulting ^FC-A^PVA scaffold was immersed in a precursor solution containing AA monomer, photoinitiator, and NaOAc for saturation. Finally, the composite was subjected to UV irradiation (365 nm) to form the interpenetrating network.

### Characterization

The morphological anisotropy and crystalline structure were analyzed using Scanning Electron Microscopy (SEM), Confocal Laser Scanning Microscopy (CLSM), Small/Wide-Angle X-ray Scattering (SAXS/WAXS), and Differential Scanning Calorimetry (DSC). Mechanical properties, including tensile strength and fatigue thresholds, were evaluated using a universal testing machine. Electrochemical quantifications, including ionic conductivity and relative permittivity, were conducted via Electrochemical Impedance Spectroscopy (EIS). The ionic thermoelectric performance was assessed by measuring the thermally induced voltage and current under controlled temperature gradients using a digital source meter. Detailed procedures and parameters are provided in the Supplementary data.

## Supplementary Material

nwag296_Supplemental_File
