# Supplementary material for: Confinement-induced giant ionic thermovoltage at minimal temperature gradients via series-integrated micro-thermoelectric cells in hierarchical hydrogels
Source: Natl Sci Rev. 2026 May 23;13(11):nwag296. doi: 10.1093/nsr/nwag296 (PMC13281098; doi:10.1093/nsr/nwag296)
Supplement: nwag296_Supplemental_File [file nwag296_supplemental_file.pdf]

Supplementary data

Confinement-induced giant ionic thermovoltage at minimal temperature gradients via series-integrated micro-thermoelectric cells in hierarchical hydrogels

Mi Fu[1,†], Yuwei Yuan[1,†], Faqi Hu[1], Liguo Xu[3], Dan Zhao[2,*] and Kan Yue[1,4,*]

[1]State Key Lab of Luminescent Materials and Devices, Guangdong Provincial Key Laboratory of Functional and Intelligent Hybrid Materials and Devices, Guangdong Basic Research Center of Excellence for Energy and Information Polymer Materials, South China Advanced Institute for Soft Matter Science and Technology, School of Emergent Soft Matter, South China University of Technology, Guangzhou 510640, China; [2]Laboratory of Organic Electronics, Department of Science and Technology, Linköping University, Norrköping 601 74, Sweden; [3]College of Light Chemical Industry and Materials Engineering, Shunde Polytechnic University, Foshan 528333, China; [4]Beijing National Laboratory for Molecular Sciences, Beijing 100190, China

***Corresponding authors.** E-mails: kanyue@scut.edu.cn; dan.zhao@liu.se

[†]Equally contributed to this work.

Contents

Experimental Section **S3**

Table S1-S5. .... **S8**

Table S1. Summary of crystallinity calculated from DSC results of $^{FC\text{-}A}$PVA-PAA-NaOAc ionic hydrogels S8
Table S2. Summary of average size of crystallites and average distance between adjacent crystallites S9
Table S3. Summary of geometric parameters used for ionic conductivity measurements in the perpendicular direction. S10
Table S4. Summary of geometric parameters used for ionic conductivity measurements in the parallel direction. S11
Table S5. Specific values and average values of thermal conductivity of $^{FC\text{-}A}$PVA-PAA-NaOAc ionic hydrogels S12

Fig. S1-S21. .... **S13**

Figure S1. TGA spectra of $^{FC\text{-}A}$PVA and $^{FC\text{-}A}$PVA-PAA-1.0M ionic hydrogels. S13
Figure S2. DSC spectra of $^{FC\text{-}A}$PVA-PAA-NaOAc ionic hydrogels with different NaOAc concentrations. S14
Figure S3. FT-IR spectra of PAA, $^{FC\text{-}A}$PVA, $^{FC\text{-}A}$PVA-PAA and $^{FC\text{-}A}$PVA-PAA-1.0M ionic hydrogels. S15
Figure S4. Pore size distribution histograms extracted from SEM images. S16
Figure S5. An in situ confocal image of the $^{FC\text{-}A}$PVA-PAA-1.0M ionic hydrogel S17
Figure S6. Nyquist plots of impedance spectra of the ionic hydrogels at different NaOAc contents in perpendicular direction. .. S18
Figure S7. Nyquist plots of impedance spectra of the ionic hydrogels at different NaOAc contents in parallel direction. S19
Figure S8. (a) Schematic and (b) real picture of the measurement setup for the i-TE conversion of the ionic hydrogels. S20
Figure S9. Variation of thermovoltage with the temperature of the $^{FC\text{-}A}$PVA-PAA-NaOAc ionic hydrogels. S21
Figure S10. Real-time temporal response of the generated thermovoltage for homogeneous PEO-PAA-NaOAc ionic hydrogel. S22
Figure S11. Variation of thermovoltage with the temperature of the $^{FC\text{-}A}$PVA-1.0M ionic hydrogels. S23
Figure S12. Time-dependent thermovoltage of the $^{FC\text{-}A}$PVA-PAA-1.0M ionic hydrogel (ΔT = 6 K) maintained for over 2 h. S24
Figure S13. Real-time temporal response of the generated thermovoltage of four parallel $^{FC\text{-}A}$PVA-PAA-1.0M samples. S25
Figure S14. CV curves of the $^{FC\text{-}A}$PVA-PAA-1.0M ionic hydrogels S26
Figure S15. Continuous output power density for 2 h with the external resistances of 900 kΩ. S27
Figure S16. Schematic illustration of “Parallel” and “Perpendicular” orientations of PVA fibrils.. .... S28
Figure S17. Summarized tensile strength, toughness, and young’s modulus of $^{FC\text{-}A}$PVA-PAA-NaOAc ionic hydrogels. S29
Figure S18. Rheological analysis of $^{FC\text{-}A}$PVA-PAA-1.0M ionic hydrogels. S30
Figure S19. Calculated total and dissipated energy and dissipated ratio of the $^{FC\text{-}A}$PVA-PAA-1.0M ionic hydrogels. S31
Figure S20. Energy dissipation and energy dissipation ratio of $^{FC\text{-}A}$PVA-PAA-1.0M ionic hydrogels over 1000 cyclic loads. S32
Figure S21. Pure shear test for cyclic fatigue test. S33

References. **S34**

Experimental Section

Chemicals and Materials

Poly(vinyl alcohol) (PVA) with average molecular weights M_w = 146-186 kDa were obtained from Sigma-Alsdrich. Acrylic acid (98.0%, AA), 2-hydroxy-4'-(2-hydroxyethoxy)-2-methylpropiophenone (98.0%, Irgacure 2959), glutaraldehyde (25% in water) and sodium acetate (99%, (NaOAc) were obtained from Adamas-beta® (Co., Ltd., China). 5-(4,6-Dichlorotriazinyl)aminofluorescein (98%, 5-DTAF) used for confocal microscope characterization was obtained from Achem-block. All materials were used directly without any purification process.

Synthesis of the $^{FC\text{-}A}$PVA-PAA-NaOAc ionic hydrogels

Poly(vinyl alcohol) (PVA, 10 g) was dissolved in deionized water (90 mL) under vigorous stirring at 100°C for 5 h to prepare a homogeneous 10 wt.% aqueous solution. Upon degassing, the 10 wt.% PVA solution was transferred into a 3D-printed polylactic acid (PLA) mold positioned atop a copper billet, with half of the billet immersed in liquid nitrogen. This configuration drives vertical ice crystal nucleation and growth, forming parallel ice columns. For ^{FC}PVA hydrogels prepared via a single freeze-casting cycle, the frozen specimen was lyophilized at -80°C and 1 Pa for 120 h after freezing, yielding an anisotropic ^{FC}PVA scaffold. The anisotropic ^{FC}PVA scaffold obtained via freeze-casting was annealed at 100°C for 90 min to yield the $^{FC\text{-}A}$PVA scaffold. Subsequently, the $^{FC\text{-}A}$PVA scaffold was immersed in a prepolymer solution containing acrylic acid (AA), photoinitiator (Irgacure 2959), and sodium acetate (NaOAc) for a minimum of 3 days to ensure complete infiltration. The swollen scaffold was transferred to a glovebox, purged with nitrogen, and photopolymerized under UV light (365 nm, 72 W) for 1 h to yield the final $^{FC\text{-}A}$PVA-PAA-NaOAc ionic hydrogels.

Thermal Characterization

Thermogravimetric analysis (TGA) measurements were performed on a Discovery TGA 2 (METTLER TOLEDO, Switzerland) with a temperature range from room temperature up to 800°C at a scanning rate of 10 °C/min under a flowing N_2 environment. Thermal transition properties were measured by a differential scanning calorimetry (DSC Discovery 2500) to determine the glass-transition temperature (T_g) at a heating/cooling rate of 10 °C/min from −30°C to 150°C.

SEM Characterization

All hydrogel samples frozen in liquid nitrogen were fractured immediately and further freeze-dried using a freeze drier (Free Zone, Labconco) to obtain the related aerogels. The fracture surfaces were then sputter-coated with platinum using an ion-sputtering device (Q150T ES plus, Quorum) and further observed with a SEM (JSM-7900F, JEOL).

Confocal Microscope Characterization

To observe the microstructures of the PVA hydrogel samples, the PVA hydrogels (20 mm (L) × 20 mm (W) × 20 μm (t)) were chemically labelled with a fluorescent dye, 5-[(4,6-dichlorotriazin-2-yl)amino]fluorescein hydrochloride (5-DTAF). The hydrogel microstructures were imaged using a confocal laser scanning microscope (LSM880).

FTIR Characterization

The Fourier-transform infrared spectroscopy (FTIR) test was performed on an infrared spectrometer (Nicolet iS 5 FTIR, Thermo Fisher Scientific Inc., U.S.A.) The sample is in the form of a thin film, and the test method is the attenuated total reflection (ATR) mode.

Mechanical Characterization

All tensile tests were performed by an electronic universal testing machine (LD22.102 instrument, LiShi, Shanghai). To avoid systematic fracture failure of the samples in the vicinity of the fixture, the hydrogels were cut into dumbbell shape with a dimension of 2×35×1.2 mm^3. A 100-N loading cell was used to perform regular tensile or cyclic tensile tests at a constant tensile rate of 100 mm min^{-1}. The toughness of the samples was calculated from the integration of the stress-strain curves in the tensile tests.

Rheological characteristics.

Rheological properties were measured by the ARES-G2 rheometer (TA Instruments, New Castle, DE, USA) equipped with an 8 mm diameter plate and a 1.0 mm plate-to-plate distance. To measure the linear viscoelastic region, the amplitude sweep rheological analyses were evaluated at a constant angular frequency of 1 Hz with the strain range of 0.01-200%. To measure the G' and G" values, the frequency sweep rheological analyses were evaluated in the linear viscoelastic region at a constant strain of 0.1% with angular frequency range of 100-0.1 rad/s. Except for temperature-related tests, all other tests were performed at 25 °C.

WAXS and SAXS Measurement

The wide-angle x-ray scattering (WAXS) test was performed on the Rigaku (HomeLab) instrument with an X-ray of λ = 0.154 nm, and the WAXS profiles were collected in the 2θ range of 5° to 60° with a speed at 5° min^{-1}. The average size of crystalline domains (D) can be calculated using the Scherrer's equation [S1]:

$$D = \frac{k\lambda}{\beta cos\theta},$$

where dimensionless shape factor (k) is set as 1 if the shape of the crystalline domain is approximated as a sphere, λ is the wavelength of x-ray diffraction, θ is the Bragg angle and β is the full width at half

maximum of the peak.

The small-angle x-ray scattering (SAXS) measurements were carried out at Xeuss 3.0 SAXS/WAXS System (Xenocs) with an X-ray of λ = 0.154 nm. The sample-to-detector distance was 1600 mm, and the exposure time was set as 180 s. The average distance between adjacent crystalline domains (L) can be calculated from the q of scattering peak (q_{max}) using the Bragg equation [S2]:

$$L = \frac{2\pi}{q_{max}}.$$

Measurement of Crystallinity

The crystallinity values of all PVA hydrogels in their dry state were quantified by a Differential Scanning Calorimetry (DSC2500, TA Instruments). All the PVA hydrogels were first chemically crosslinked with excessively glutaraldehyde (5 mL glutaraldehyde solution, 0.5 mL hydrochloric acid with the concentration of 38 wt%, and 105 mL deionized), in order to fix the amorphous polymer chains before air-drying, thus no further crystallization will occur during the drying process.

All the hydrogel samples were first dried in a drying oven at 40°C for 2 days and the total mass was measured as m_{dry} before the DSC test. For the DSC test, the air-dried samples were heated up from 30°C to 250°C at a rate of 10 °C min^{-1}, under a nitrogen flow of 50 mL min^{-1}. The narrow peak present in the heat flow curve ranging from 180°C to 230°C corresponds to melting of the PVA crystalline domains. The enthalpy could be calculated by integrating endothermic transition of the melting area of the crystalline domains of the dried hydrogels ($H_{crystalline}$). Thus, the mass of the crystalline domains ($m_{crystalline}$) could be calculated as [S3]:

$$m_{crystalline} = \frac{H_{crystalline} \times m_{dry}}{H^0_{crystalline}},$$

where $H^0_{crystalline}$ = 138.6 J g^{-1} is the enthalpy of melting of 100% crystalline PVA. Therefore, the crystallinity in the dry state (X_{dry}) and the swollen state ($X_{swollen}$) can be calculated as:

$$X_{dry} = \frac{m_{crystalline}}{m_{dry}} \times 100\%,$$

$$X_{swollen} = \frac{X_{dry}}{1-\phi_{water}} \times 100\%.$$

Fatigue Tests

The hydrogel was cut into rectangular samples of 40 mm length and 10 mm width with a notch of 2 mm in the middle, and the notched samples were subjected to cyclic tensile tests under different stretches using an electronic universal testing machine. The changes in the length of the crack were recorded by a digital microscope (AM4815, Dino-Lite), and the average distance of crack extension in each tensile cycle was calculated (dc/dN). The stress- stretch curves of the unnotched samples under the same stretch conditions

were measured and the strain energy density $W(\lambda, N)$ at the Nth tensile cycle was calculated [S3-S5]:

$$W(\lambda, N) = \int_1^\lambda S\, d\lambda,$$

where S represents stress and λ represents stretch. The energy release rate (G) of the notched sample in the Nth tensile cycle can be calculated as:

$$G(\lambda, N) = 2k(\lambda) \cdot c(N) \cdot W(\lambda, N),$$

where c represents the crack length and k represents the function of the applied stretch (λ), which can be calculated empirically as:

$$k = 3 \cdot \lambda^{-1/2}.$$

By varying the stretch applied in the cyclic tensile test, a curve can be obtained for the relationship between the average distance of crack extension (dc/dN) and the rate of energy release (G) in each cycle. Then, based on the linear extrapolation of the relationship curves of dc/dN and G, the intercept with the horizontal coordinate is found and the fatigue threshold (Γ_0), which is the energy release rate corresponding to the time when fatigue crack extension just does not occur. To verify the authenticity of the fatigue threshold obtained by linear extrapolation, the notched sample is subjected to 5000 cyclic tensile tests under the condition of this energy release rate, and the expansion of fatigue cracks is recorded by digital microscope at the same time.

Electrochemical Impedance and Dielectric Analysis

The electrical characterization of the ionic hydrogels was performed on an electrochemical workstation (CHI660e, CH Instruments, USA). The impedance is measured by the EIS method. The initial voltage was the measured open voltage; the frequency ranged from 1 Hz to1 MHz; and the AC amplitude was 0.01 V. The ionic conductivity was measured using thin film samples with dimensions of 10 × 10 × 1.2 mm^3. Two ends of the sample were clamped with platinum steel discs, leaving 1 mm ionic conduct distance (L) in the middle. Then the Nyquist plot of the example gel can be obtained by this method. To use the formula to calculate the ionic conductivity:

$$\sigma = L/RS,$$

where σ is the ionic conductivity, L is the thickness of the sample, R is bulk resistance of the sample obtained from the Nyquist plot and area of the sample.

Calculation of the Relative Permittivity (Revealing Intrinsic Polarization Ability)

Firstly, the real part of the complex capacitance, $C'(f)$, which represents the charge storage capability, was calculated from the electrochemical impedance spectroscopy (EIS) data using the following relationship[S6]:

$$C'(f) = -\frac{Z''(f)}{2\pi f[Z'(f)^2 + Z''(f)^2]},$$

where f is the frequency (Hz), $Z'(f)$ and $Z''(f)$ are the real and imaginary parts of the complex impedance Z (Ω), respectively.

To eliminate geometric effects and compare the intrinsic polarization ability of the material in different directions, the relative permittivity (dielectric constant) was calculated. For a parallel-plate capacitor geometry, the relative permittivity $\varepsilon_r'(f)$ is derived from the measured capacitance $C'(f)$ [S7]:

$$\varepsilon_r'(f) = \frac{C'(f) \cdot L}{\varepsilon_0 \cdot S},$$

where L is the thickness (m) of the sample between electrodes, S is the electrode contact area (m²), $\varepsilon_0 = 8.854 \times 10^{-12}$ F · m^{-1} is the vacuum permittivity.$\varepsilon_r'(f)$ is the frequency-dependent real part of the relative permittivity.

Thermal-electrical Characterization

The short-circuit voltage and short-circuit current of the sample under the given temperature difference are measured by the digital source meter (2450, Keithley Instruments, Inc., USA). The ionic hydrogels sample are made into a block of 15×15×1.2 mm^3 and the upper and lower surfaces are covered with the platinum sheet of 0.5 mm thick.

Thermal Conductivity Characterization

The thermal conductivity of the sample was measured by the transient hot-wire method using a thermal conductivity instrument (TC 3000E, XIATECH, China). The experimental voltage is 1V, and the acquisition time is 10 seconds. The each inongel sample was made into a block of 20×30×1.2 mm^3, two blocks are required for each concentration from 0.3 M to 1.3 M. The measurement was repeated at least 5 times for 3 times, and the average value is determined.

Table S1-S5.

Table S1. Summary of crystallinity calculated from DSC results of $^{FC\text{-}A}$PVA-PAA-NaOAc ionic hydrogels with different NaOAc concentrations.

Sample	Crystallinity (%)
$^{FC\text{-}A}$PVA-PAA	0
$^{FC\text{-}A}$PVA-PAA-0.3M	9.3
$^{FC\text{-}A}$PVA-PAA-0.5M	10.0
$^{FC\text{-}A}$PVA-PAA-0.7M	15.7
$^{FC\text{-}A}$PVA-PAA-1.0M	19.3
$^{FC\text{-}A}$PVA-PAA-1.3M	16.4

Table S2. Summary of average size of crystallites and average distance between adjacent crystallites of $^{FC-A}$PVA-PAA-NaOAc ionic hydrogels with different NaOAc concentrations.

Sample	Crystallite size (nm)	Distance between crystallites (nm)
$^{FC-A}$PVA-PAA	1.301	15.2
$^{FC-A}$PVA-PAA-0.3M	1.488	10.6
$^{FC-A}$PVA-PAA-0.5M	1.488	10.5
$^{FC-A}$PVA-PAA-0.7M	1.817	10.1
$^{FC-A}$PVA-PAA-1.0M	1.824	10.1
$^{FC-A}$PVA-PAA-1.3M	1.803	10.1

Table S3. Summary of geometric parameters used for ionic conductivity measurements of $^{FC\text{-}A}$PVA-PAA-NaOAc hydrogels with varying NaOAc concentrations in the perpendicular direction.

**Perpendicular-sample**	***L* (mm)**	***S* (mm^2)**	***R* (Ω)**	**σ_i (mS cm^{-1})**
0.3M	1.434	100	206.1	0.696
	1.434	100	208.9	0.686
	1.477	100	148.5	0.995
0.5M	1.633	100	148.0	1.103
	1.594	70.0	159.4	1.429
	1.483	100	138.2	1.073
0.7M	1.461	100	87.73	1.665
	1.479	100	82.52	1.792
	1.501	100	82.21	1.826
1.0M	1.266	100	104.9	1.207
	1.166	33.6	236.5	1.470
	1.156	42.5	235.1	1.157
1.3M	1.283	100	155.0	0.828
	1.240	100	144.0	0.861
	1.183	68.7	169.0	1.019

Table S4. Summary of geometric parameters used for ionic conductivity measurements of $^{FC\text{-}A}$PVA-PAA-NaOAc hydrogels with varying NaOAc concentrations in the parallel direction.

**Parallel-sample**	***L* (mm)**	***S* (mm^2)**	***R* (Ω)**	**σ_i (mS cm^{-1})**
0.3M	2.363	13.89	482.2	3.528
	1.509	11.53	342.9	3.817
	1.988	11.55	480.0	3.586
0.5M	2.044	13.05	224.1	6.989
	1.949	12.56	213.9	7.255
	1.949	12.56	212.9	7.289
0.7M	1.715	11.26	204.2	7.459
	1.715	11.26	203.7	7.477
	1.965	11.22	225.7	7.760
1.0M	1.822	11.03	224.6	7.355
	1.822	11.03	226.0	7.309
	1.465	10.64	211.1	6.520
1.3M	1.628	9.02	249.9	7.222
	2.135	9.08	317.8	7.399
	1.793	9.09	362.0	5.449

Table S5. Specific values and average values of thermal conductivity of $^{FC-A}$PVA-PAA-NaOAc ionic hydrogels with different NaOAc concentrations in perpendicular direction.

**Number**	**Thermal Conductivity (W/m*K)**
0M-1	0.4555
0M-2	0.4603
0M-3	0.4592
0M-AVG	**0.4583**
0.3M-1	0.4822
0.3M-2	0.4855
0.3M-3	0.4801
0.3M-AVG	**0.4826**
0.5M-1	0.4823
0.5M-2	0.4821
0.5M-3	0.4821
0.5M-AVG	**0.4822**
0.7M-1	0.4730
0.7M-2	0.4727
0.7M-3	0.4722
0.7M-AVG	**0.4726**
1.0M-1	0.4837
1.0M-2	0.4838
1.0M-3	0.4855
1.0M-AVG	**0.4843**
1.3M-1	0.4761
1.3M-2	0.4746
1.3M-3	0.4758
1.3M-AVG	**0.4755**

Fig. S1-S21.

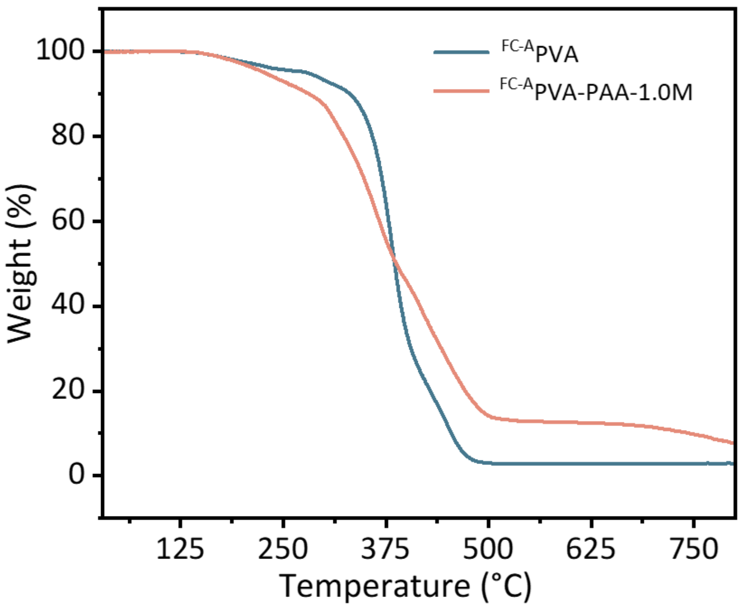

Figure S1. TGA spectra of FC-APVA and FC-APVA-PAA-1.0M ionic hydrogels.

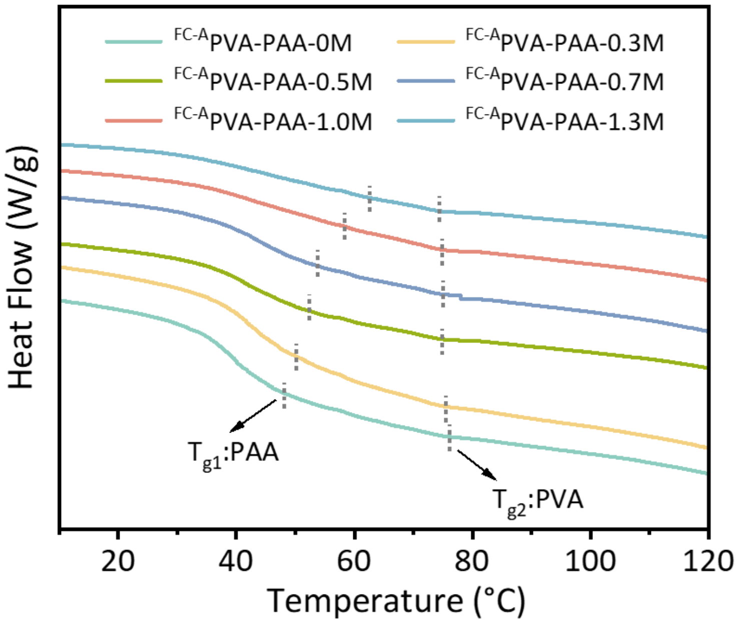

Figure S2. DSC spectra of $^{FC\text{-}A}$PVA-PAA-NaOAc ionic hydrogels with different NaOAc concentrations.

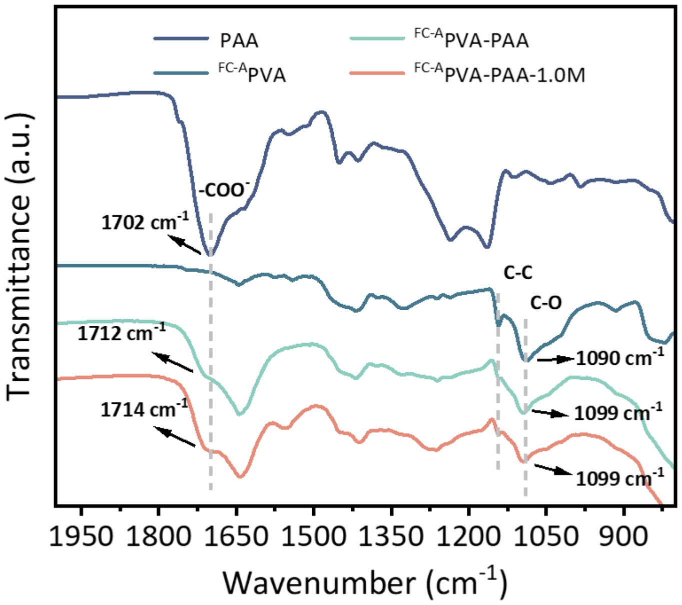

Figure S3. FT-IR spectra of PAA, $^{FC\text{-}A}$PVA, $^{FC\text{-}A}$PVA-PAA and $^{FC\text{-}A}$PVA-PAA-1.0M ionic hydrogels.

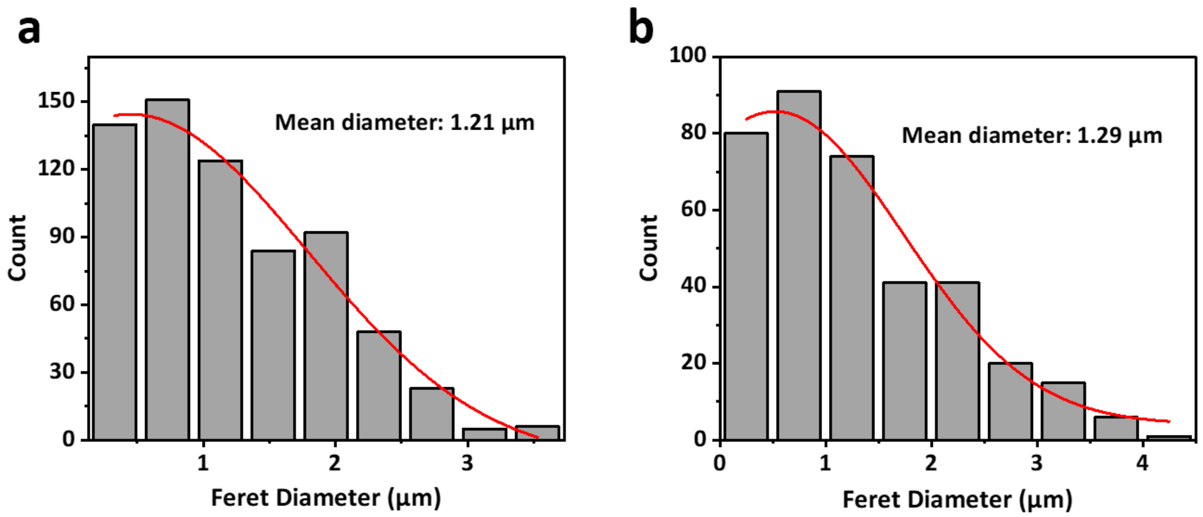

Figure S4. Pore size distribution histograms extracted from SEM images. (a) The pure $^{FC\text{-}A}$PVA skeleton exhibiting a mean Feret diameter of 1.21 µm. (b) The $^{FC\text{-}A}$PVA-PAA-1.0M ionic hydrogel exhibiting a mean Feret diameter of 1.29 µm.

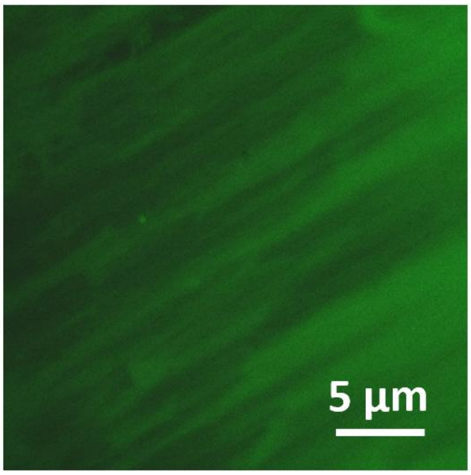

Figure S5. An in situ confocal image of the $^{FC\text{-}A}$PVA-PAA-1.0M ionic hydrogel added with fluorescein sodium salt as a fluorescent marker.

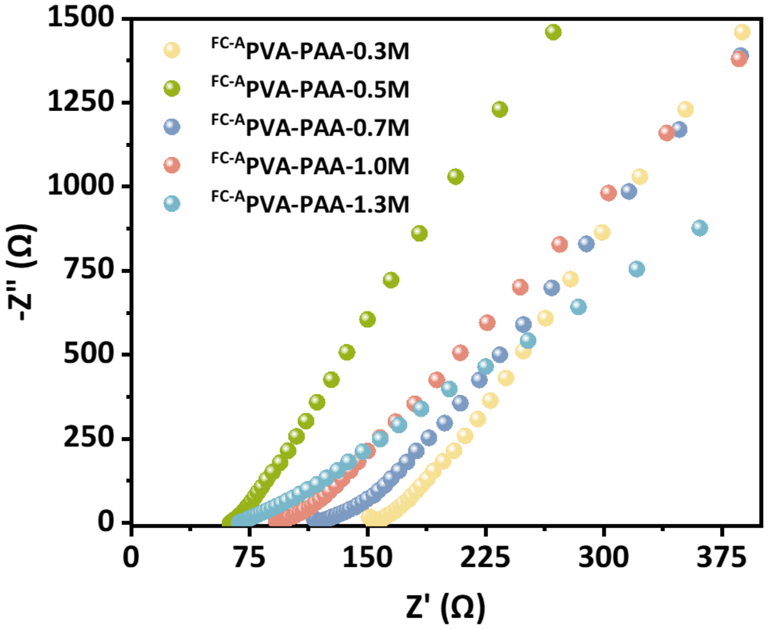

Figure S6. Nyquist plots of impedance spectra of the ionic hydrogels at different NaOAc contents in perpendicular direction.

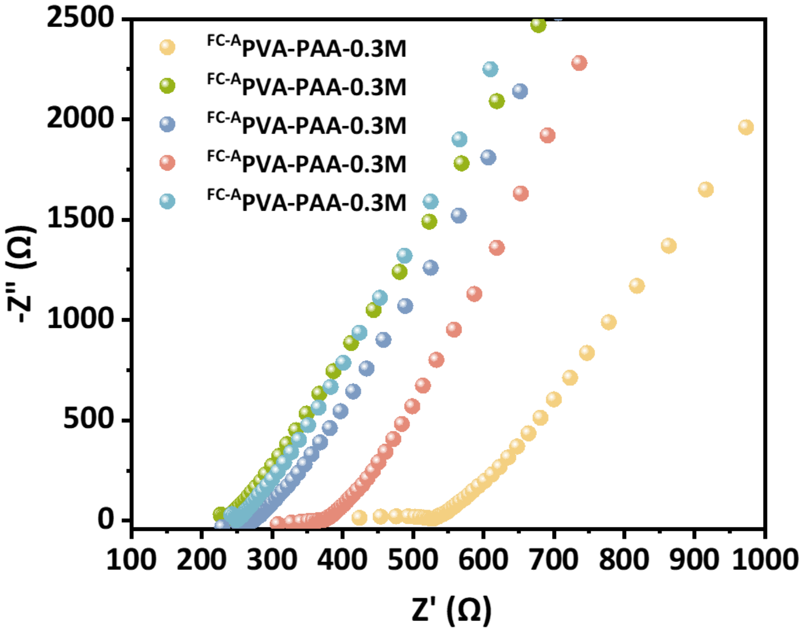

Figure S7. Nyquist plots of impedance spectra of the ionic hydrogels at different NaOAc contents in parallel direction.

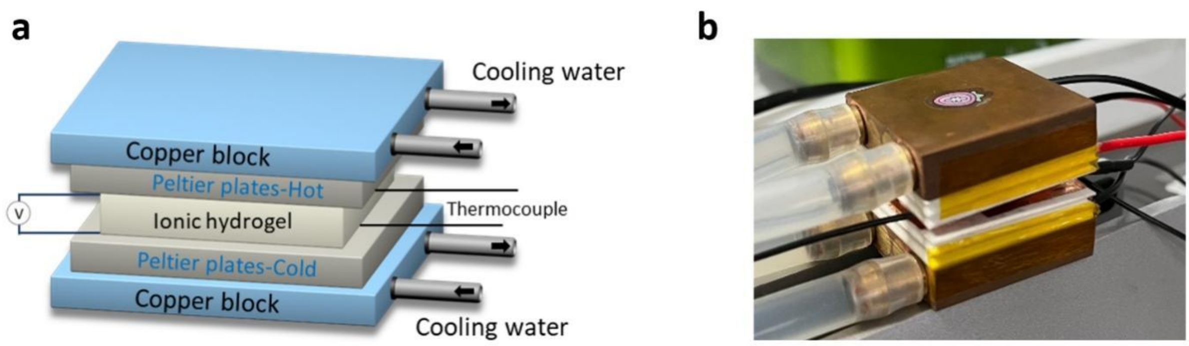

Figure S8. (a) Schematic and (b) real picture of the measurement setup for the i-TE conversion of the ionic hydrogels.

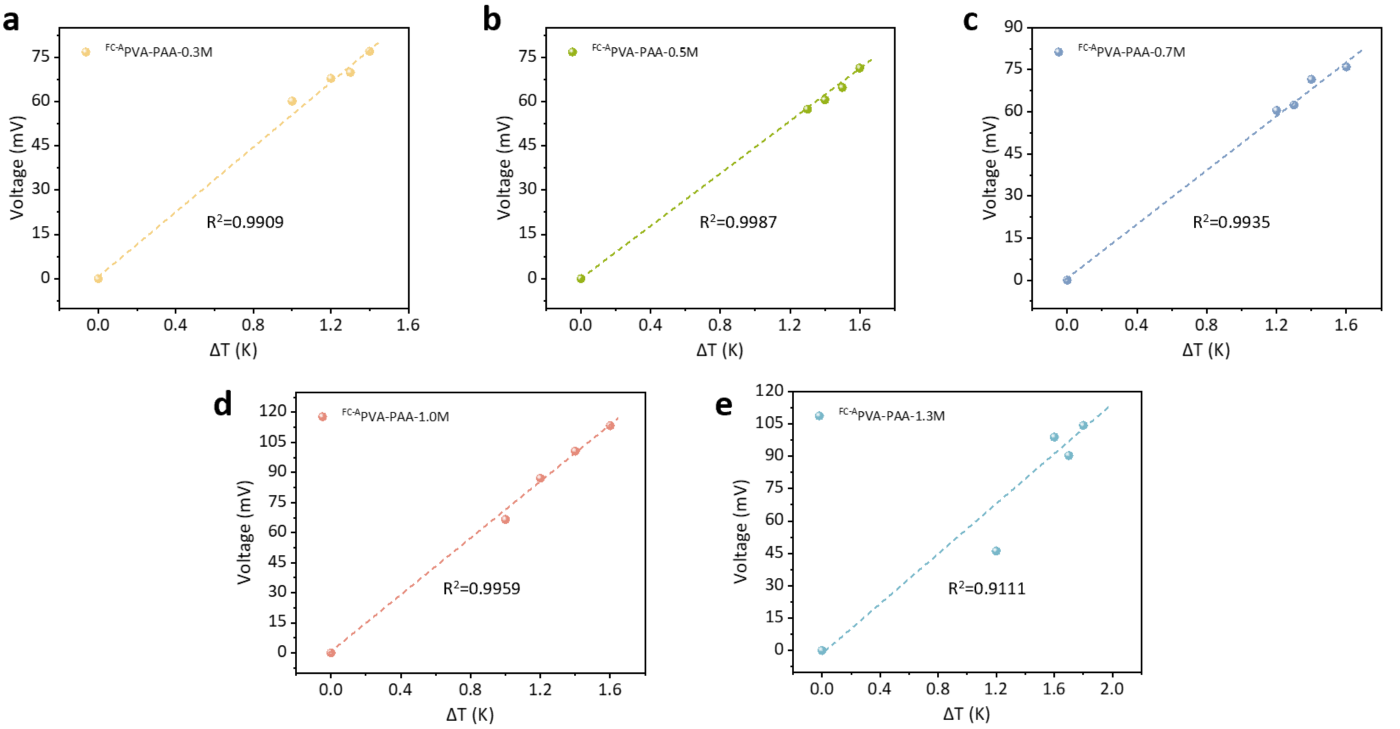

Figure S9. Variation of thermovoltage with the temperature of the $^{FC\text{-}A}$PVA-PAA-NaOAc ionic hydrogels with different NaOAc concentrations at small Δ*T*.

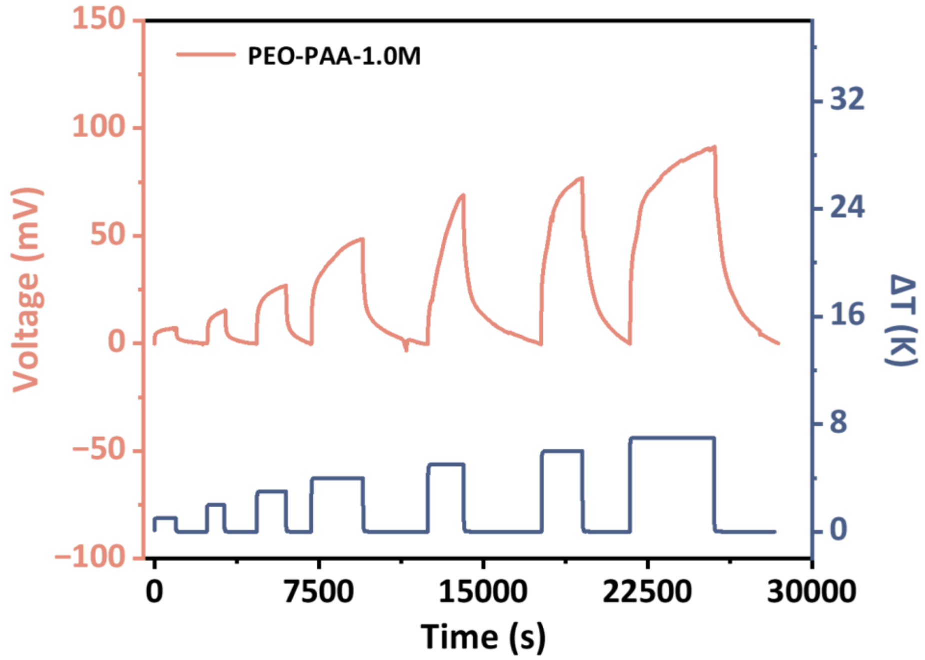

Figure S10. Real-time temporal response of the generated thermovoltage and the applied temperature difference for homogeneous PEO-PAA-NaOAc ionic hydrogel.

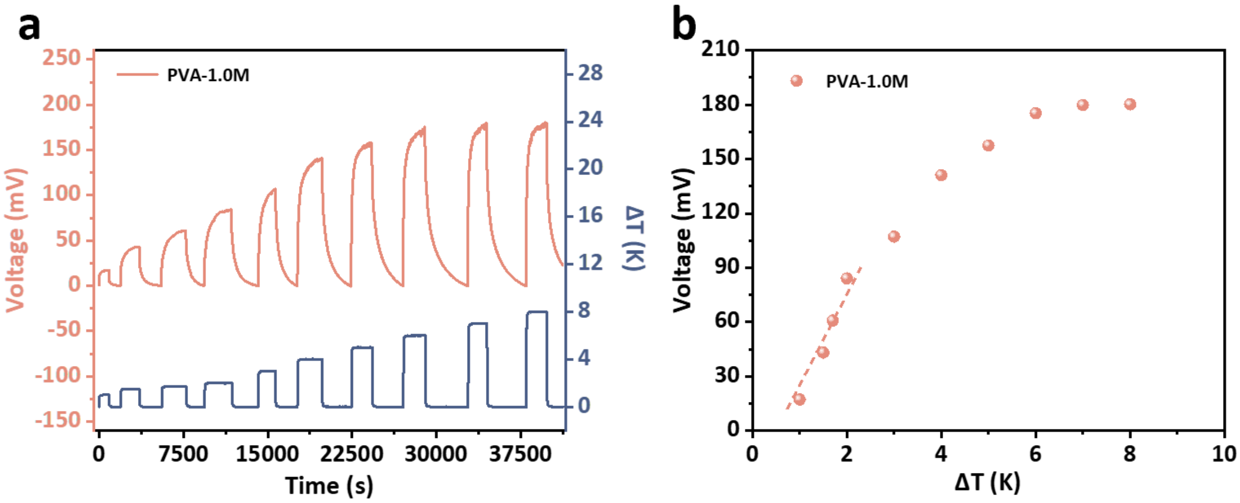

Figure S11. (a) Thermovoltage of the $^{FC\text{-}A}$PVA-1.0M ionic hydrogel. (b) Variation of thermovoltage with the temperature of the $^{FC\text{-}A}$PVA-1.0M ionic hydrogels.

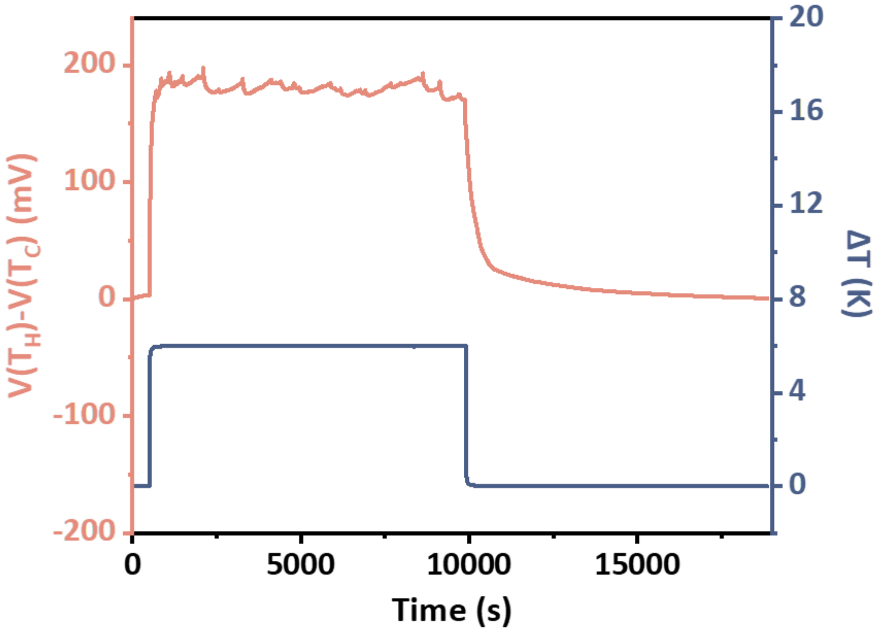

Figure S12. Time-dependent thermovoltage profile of the $^{FC\text{-}A}$PVA-PAA-1.0M ionic hydrogel under a constant temperature difference (ΔT = 6 K) maintained for over 2 hours.

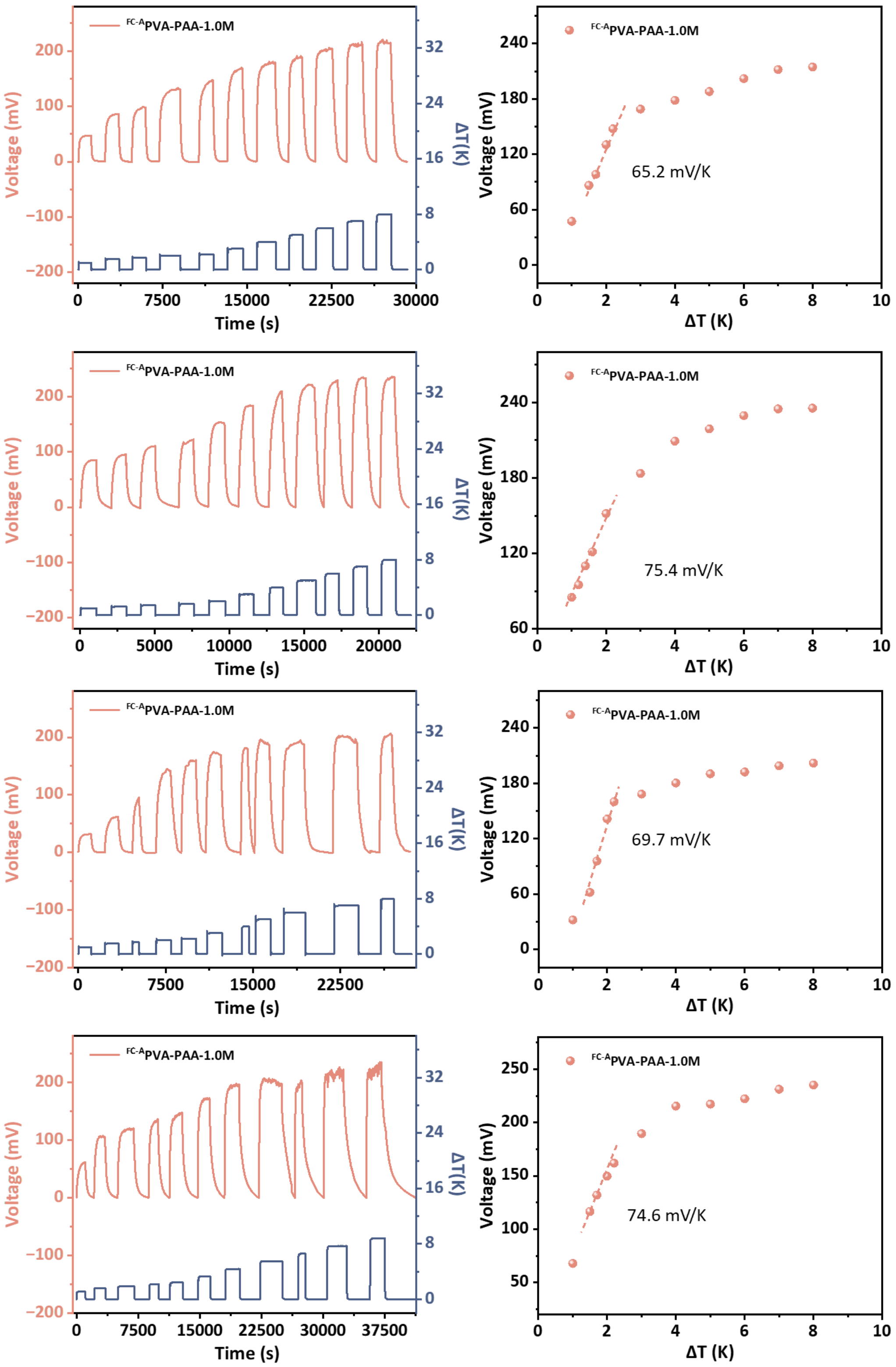

Figure S13. Real-time temporal response of the generated thermovoltage and the applied temperature difference of four parallel $^{FC\text{-}A}$PVA-PAA-1.0M samples.

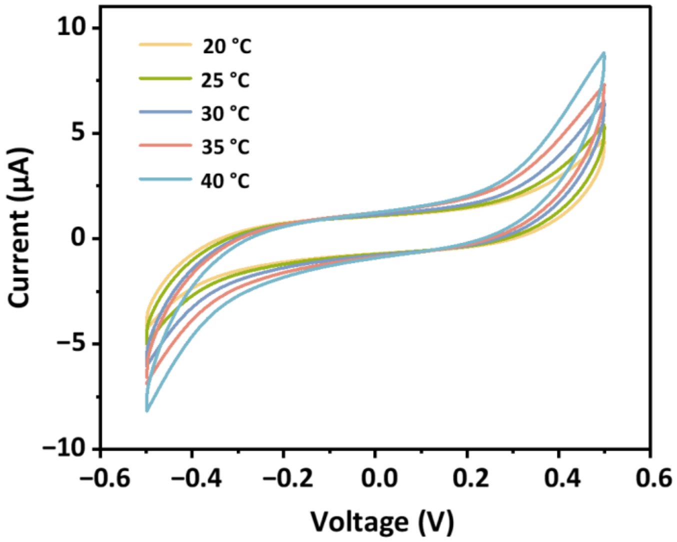

Figure S14. Cyclic voltammetry curves of the $^{FC\text{-}A}$PVA-PAA-1.0M ionic hydrogels measured at different temperatures with a scan rate of 50 mV s^{-1}. The measurements were conducted using a symmetric two-electrode configuration (Pt/hydrogel/Pt) under isothermal conditions ($\Delta T = 0$ K).

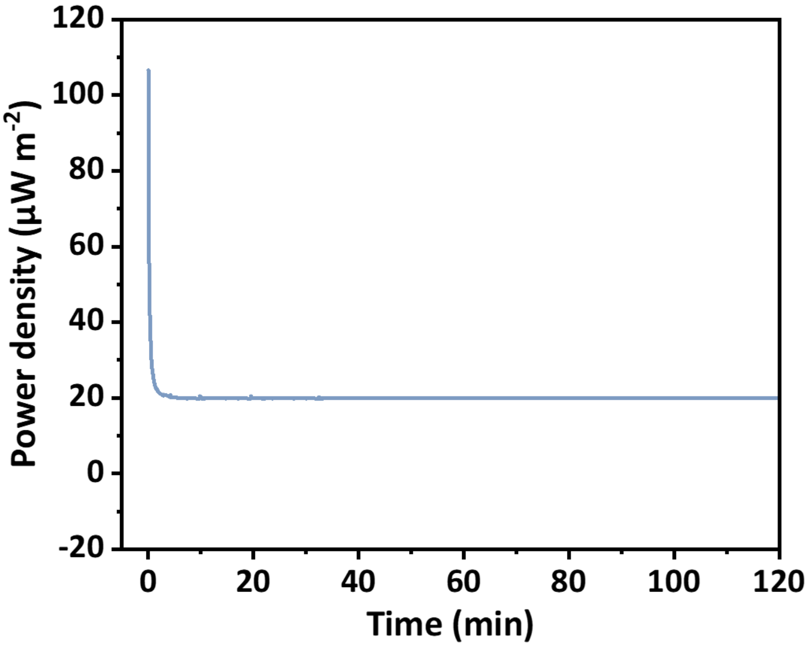

Figure S15. Continuous output power density for 2 h with the external resistances of 900 kΩ.

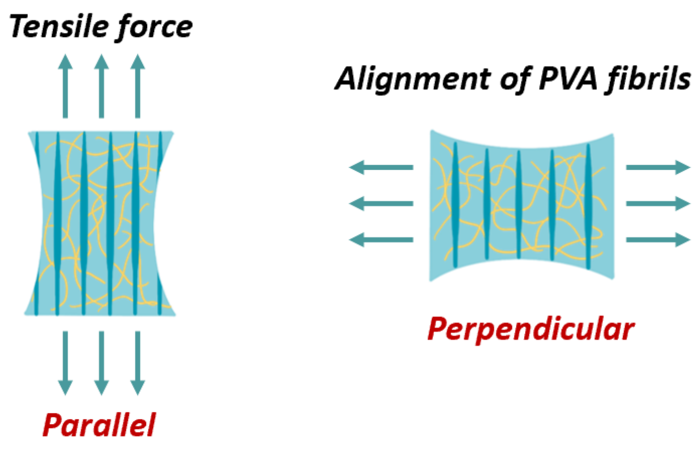

Figure S16. Schematic illustration of "Parallel" and "Perpendicular" orientations relative to the direction of the applied tensile force and the alignment of PVA fibrils.

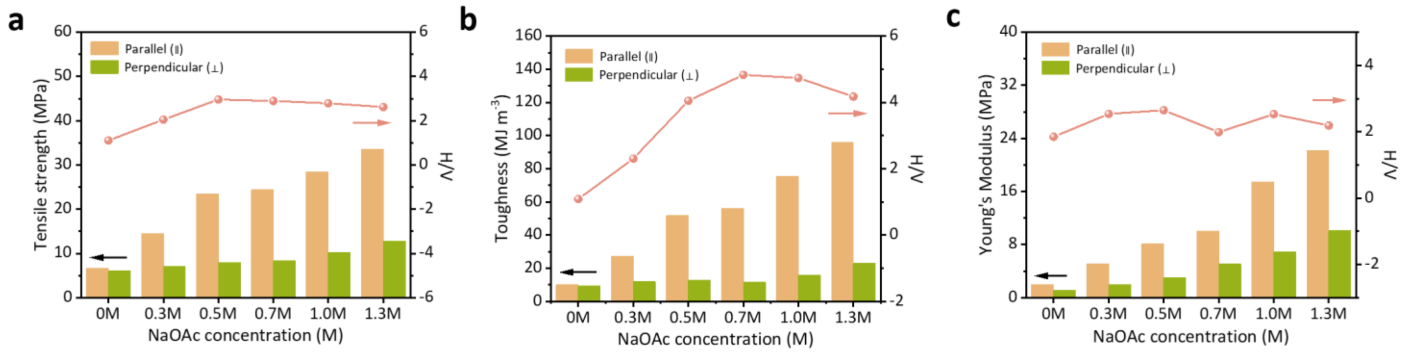

Figure S17. Summarized (a) tensile strength, (b) toughness, and (c) young's modulus of $^{FC\text{-}A}$PVA-PAA-NaOAc ionic hydrogels with different NaOAc concentrations in parallel and perpendicular directions, along with their anisotropy as reflected by H/V values in the two directions.

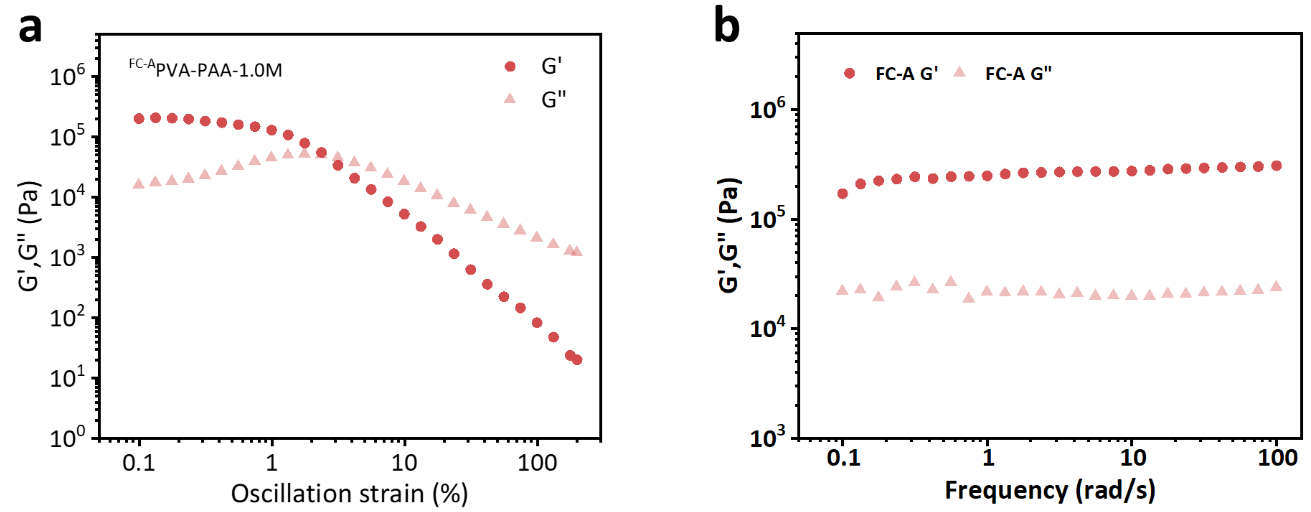

Figure S18. (a) Amplitude-sweep rheological analysis of the $^{FC\text{-}A}$PVA-PAA-1.0M ionic hydrogels. (b) Frequency-sweep rheological analysis of $^{FC\text{-}A}$PVA-PAA-1.0M ionic hydrogels.

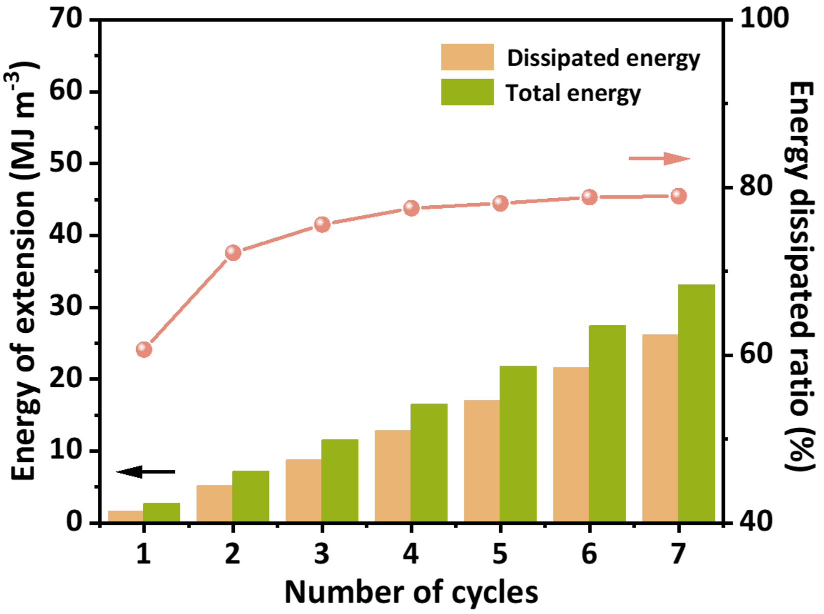

Figure S19. Calculated total and dissipated energy and dissipated ratio of the $^{FC\text{-}A}$PVA-PAA-1.0M ionic hydrogels.

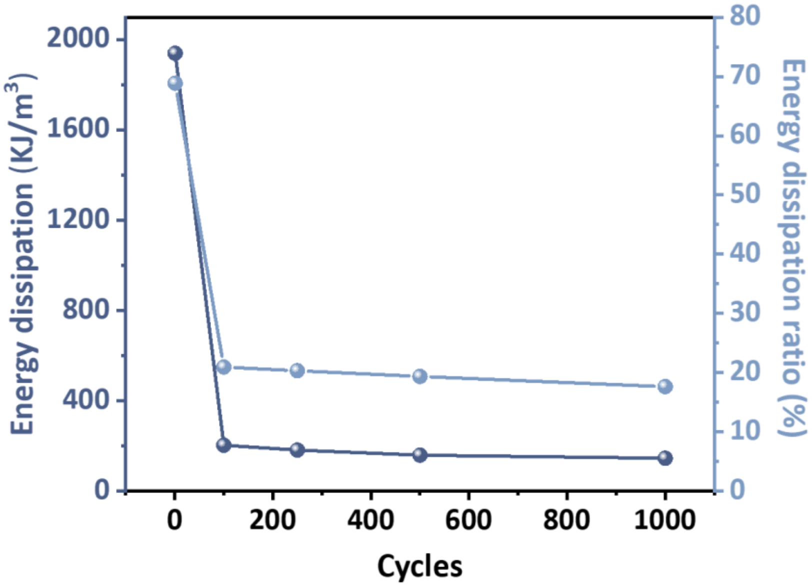

Figure S20. Energy dissipation and energy dissipation ratio of $^{FC\text{-}A}$PVA-PAA-1.0M ionic hydrogels over 1000 cyclic loads.

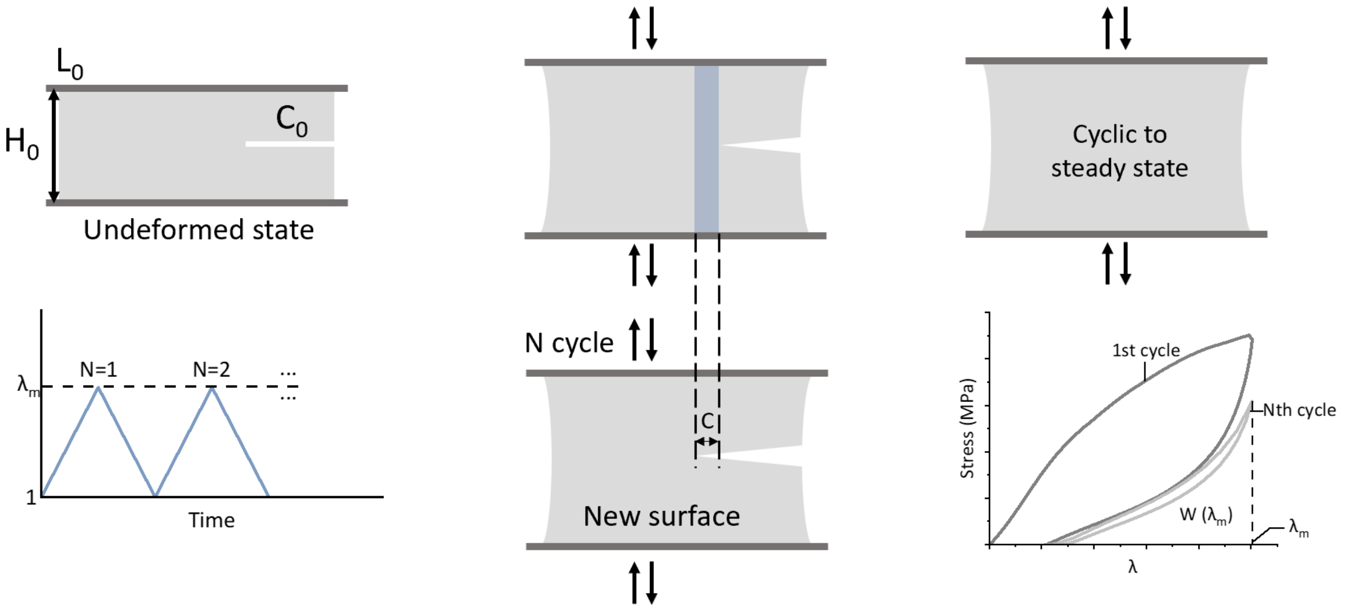

Figure S21. Pure shear test for cyclic fatigue test. From the fatigue resistance curve (*Δc/ΔN* versus *G*), one can obtain the fatigue threshold G_0 below which the crack does not grow.

References.

[S1] Liang X, Chen G, Lin S *et al.* Anisotropically fatigue-resistant hydrogels. *Adv Mater* 2021; **33**: 2102011.

[S2] Yuan F, Zhang XX, Wu K *et al.* Damping chitin hydrogels by harnessing insect-cuticle-inspired hierarchical structures. *Cell Rep Phys Sci* 2023; **4**: 101644.

[S3] Wu Y, Zhang Y, Wu H *et al.* Solvent-exchange-assisted wet annealing: a new strategy for superstrong, tough, stretchable, and anti-fatigue hydrogels. *Adv Mater* 2023; **35**: 2210624.

[S4] Hua M, Wu S, Ma Y *et al.* Strong tough hydrogels via the synergy of freeze-casting and salting out. *Nature* 2021; **590**: 594–9.

[S5] Li W, Li L, Zheng S *et al.* Recyclable, healable, and tough ionogels insensitive to crack propagation. *Adv Mater* 2022; **34**: 2203049.

[S6] Taberna PL, Simon P, Fauvarque JF. Electrochemical Characteristics and Impedance Spectroscopy Studies of Carbon-Carbon Supercapacitors. *J Electrochem Soc* 2003; **150**: A292.

[S7] Lunkenheimer P, Bobnar V, Pronin AV *et al.* Origin of apparent colossal dielectric constants. *Phys Rev B* 2002; **66**: 052105.
